# Inhibiting ribosomal RNA synthesis in *C. elegans* protects against reductive stress during de novo fatty acid synthesis deficiency

**DOI:** 10.1093/genetics/iyag214

**Published:** 2026-08-11

**Authors:** Jen F Rotti, Fasih Ahsan, Nicole L Stuhr, Sinclair Emans, Alexander Soukas, Armen Yerevanian

**Affiliations:** Department of Medicine, Diabetes Unit and Center for Genomic Medicine, Massachusetts General Hospital, Boston, MA 02114, United States; Department of Medicine, Diabetes Unit and Center for Genomic Medicine, Massachusetts General Hospital, Boston, MA 02114, United States; Program in Biological and Biomedical Sciences, Division of Medical Sciences, Harvard Medical School, Harvard University, Boston, MA 02115, United States; Broad Institute of Harvard and MIT, Cambridge, MA 02142, United States; Department of Medicine, Diabetes Unit and Center for Genomic Medicine, Massachusetts General Hospital, Boston, MA 02114, United States; Broad Institute of Harvard and MIT, Cambridge, MA 02142, United States; Department of Medicine, Diabetes Unit and Center for Genomic Medicine, Massachusetts General Hospital, Boston, MA 02114, United States; Program in Biological and Biomedical Sciences, Division of Medical Sciences, Harvard Medical School, Harvard University, Boston, MA 02115, United States; Department of Medicine, Diabetes Unit and Center for Genomic Medicine, Massachusetts General Hospital, Boston, MA 02114, United States; Broad Institute of Harvard and MIT, Cambridge, MA 02142, United States; Department of Medicine, Diabetes Unit and Center for Genomic Medicine, Massachusetts General Hospital, Boston, MA 02114, United States

**Keywords:** *C. elegans*, reductive stress, biguanides, longevity, ribosomal RNA (rRNA), nucleolar stress, WormBase

## Abstract

Reductive stress has remained underappreciated as a significant disrupter of redox homeostasis. Recent studies have begun to link the accumulation of NADH and NADPH to the development and progression of metabolic diseases such as cancer, cardiac disease, and diabetes. In this study, we use the nematode *Caenorhabditis elegans* to examine the phenomenon of catastrophic reductive death caused by combined biguanide treatment and *fasn-1* deficiency. This process of synergistic biguanide-induced reductive stress correlates with the activation of hypodermal stress response genes and aberrant alternations in the nucleolar morphology of hypodermal cells. Interestingly, we find that loss-of-function and RNAi-based knockdown of the catalytic RNA exosome subunit *crn-3* significantly protects against reductive death. RNAi knockdown of multiple other genes involved in rRNA synthesis recapitulate this phenotype. We postulate that this reversal of reductive death can be attributed to impaired ribosomal RNA biogenesis that promotes tolerance of accumulated reducing equivalents Nicotinamide Adenine Dinucleotide Phosphate (NADPH) and Nicotinamide Adenine Dinucleotide (NADH) while also preventing the accumulation of Glutathione (GSH) by potentially activating downstream signaling pathways. Notably, we identify a downstream nuclear RNAi pathway that is activated by phenformin in a *fasn-1-*dependent manner and is also activated upon disruption of rRNA processing. Overall, we identify a novel mechanism by which pathologic states of reductive stress-related diseases could be ameliorated.

## Introduction

Redox reactions are essential for the maintenance of organismal health via cell signaling, gene regulation, and metabolite generation (Xiao et al. 2018; Sies et al. 2024). NADH/NAD+, NADPH/NADP+, and GSH/GSSG are the 3 major electron carriers that act as substrates for mitochondrial and cytoplasmic redox reactions, powering critical biological processes (Xiao et al. 2018). An imbalance of oxidant and antioxidant species can lead to cellular dysfunction and adverse health outcomes (Xiao et al. 2018; Sies et al. 2024). Redox signaling during development is particularly important as early-life ROS signaling can cause epigenetic changes that promote stress resistance and longevity later in life (Bazopoulou et al. 2019). It is therefore important to understand the mechanisms through which detrimental redox stress can be mitigated.

Reductive stress—the imbalance of redox species in favor of a reduced state—has been shown to be involved in the pathology of several diseases. For example, in vivo administration of the antioxidants *N*-acetylcysteine or vitamin E accelerated the progression of lung cancer in mice, possibly by inhibiting the expression of the tumor suppressor p53, which is typically activated by oxidative stress (Sayin et al. 2014). Overexpression of molecular chaperone CryAB causes reductive stress that can accelerate cardiac hypertrophy but is reversed upon inhibition of glucose-6-phosphate dehydrogenase (G6pd), linking NADPH production via the pentose phosphate pathway to cardiac disease (Rajasekaran et al. 2011). In addition, increased glutathione peroxidase activity has also been shown to cause insulin resistance in mice (McClung et al. 2004). Thus, identifying ways to prevent reductive stress could provide crucial insight into disease pathology and potential therapeutic interventions.

Metformin, the most widely prescribed anti-type 2 diabetes medication, is a member of a class of drugs called biguanides that have been shown to alter cellular metabolism, resulting in extended health span and lifespan in a wide variety of model organisms including invertebrate worms, mice, and nonhuman primates (Soukas et al. 2019; Yang et al. 2024). Investigation into the genetic mechanisms through which biguanides extend lifespan is ongoing.

Recent work in our lab highlights the importance of maintaining redox homeostasis upon biguanide administration. Our recent studies have suggested that phenformin, a potent sister biguanide to metformin, significantly alters redox homeostasis in the invertebrate nematode *C. elegans* by enhancing the reductive ratio of NADH/NAD+, NADPH/NADP+, and GSH/GSSG couples (Ahsan et al. 2026). We found that de novo lipogenesis, the major organismal fatty acid biosynthetic process, functions as a critical brake to prevent unchecked reductive stress induced by multiple NADPH-accumulating paradigms (Ahsan et al. 2026).

Fatty acid synthesis is the major consumer of NADPH within *C. elegans* and other eukaryotes. Notably, 14 molecules of NADPH are oxidized into NADP+ in the FASN-1-catalyzed conversion of malonyl-CoA into palmitate. Inhibition of essential de novo fatty acid synthesis genes *fasn-1* and *pod-2* results in a toxic accumulation of NADPH, NADH, and GSH in animals treated with typically lifespan-extending doses metformin or phenformin; a synthetic lethal effect that is not rescued upon palmitate end-product supplementation (Ahsan et al. 2026). Supplementation with malic acid or glucose similarly promotes NADPH accumulation and phenocopies this synthetic lethal phenotype when fatty acid biosynthesis is impaired (Ahsan et al. 2026). To prevent reductive stress, *C. elegans* and mammalian cellular models may increase de novo fatty acid synthesis as a detoxifying strategy (Ahsan et al. 2026). These findings emphasize the importance of fatty acid synthesis in maintaining redox homeostasis when challenged with excessive reducing power caused by biguanides and other pharmacological and nutritional interventions. The pathology of phenformin-induced reductive stress remains unclear. In this paper, we aim to understand how organisms may protect themselves against reductive stress.

Emerging studies have begun to recognize the role of the nucleolus in cellular responses to stress. This subnuclear compartment is essential for ribosome biogenesis. The nucleolus stores ribosomal DNA (rDNA), which is transcribed to generate the ribosomal RNAs (rRNAs) that form the structural core of the small and large ribosomal subunits (González-Arzola 2024). In response to starvation, rRNA synthesis is reduced in *C. elegans* and mammalian cell models to potentially conserve energy (Wu et al. 2018; Lu et al. 2025; Shovon et al. 2025). Additionally, inhibiting RNA polymerase I activity has been shown to protect against toxicities associated with late-in-life administration of metformin in *C. elegans*, potentially by protecting metabolic plasticity and energetic homeostasis (Sharifi et al. 2024). Thus, inhibiting rRNA synthesis may aid in the adaption and resistance to stress.

Performing a high-throughput RNAi genetic screen in *C. elegans*, we demonstrate that phenformin-induced reductive stress is associated with altered nucleolar morphology and that activation of a nucleolar stress response pathway increases tolerance of reducing equivalent accumulation. These findings highlight targeting rRNA biogenesis as a potential therapeutic intervention for reductive stress.

## Methods

### *E. coli* strains and maintenance

The *E. coli* strain used for general worm maintenance and non-RNAi experiments was OP50-1. Cultures of OP50-1 were grown in LB broth for 15 to 24 h at 37 °C without shaking and were seeded onto nematode growth medium (NGM) plates. In general, plates were used within 3 to 7 d of seeding. All RNAi experiments were conducted using *E. coli*  HT115 (DE3) cells with L4440 used as a plasmid vector. RNAi bacterial cultures were grown in LB broth supplemented with carbenicillin for 15 to 24 h at 37 °C with shaking. Cultures were pelleted via centrifugation and resuspended at a final 5× concentration. Bacterial cultures were seeded directly onto NGM plates containing 200 µg/mL carbenicillin and 5 mM isopropyl-β-D-thiogalactopyranoside (IPTG). Plates were seeded at least 1 d prior to experiments, ensuring sufficient IPTG induction, and were used within 5 d of seeding. The L4440 vector was used as an empty vector (EV) control in RNAi experiments to account for any inherent effects caused by the presence of the plasmid backbone. Additionally, a nontargeting RNAi clone was created by inserting the DNA sequence for the hygromycin resistance gene HygR—which is not endogenously expressed by *C. elegans—*into the L4440 multiple cloning site to account for the inherent effects of dsRNA and the RNAi delivery system.

### Nematode strains and maintenance

*C. elegans* strains were maintained by feeding OP50-1 bacteria ad libitum on NGM agar plates at 20 °C in a temperature- and humidity-controlled Thermo Scientific Forma incubator, unless otherwise noted (Brenner 1974). Ancestral vials for each strain were freshly thawed every 3 to 4 mo to prevent genetic drift in existing cultures. The strains used can be found in the below table (Table 1).

**Table 1.** Strains.

Strain	Genotype	Notes
N2	Wild-type	Ordered from CGC
MGH770	osIs158[eft-3p::AtTIR1(F79G)::mRuby] II; [GFP::AID*::3x-FLAG::fasn-1]	Constructed for this paper
MGH887	*crn-3(ok2269)*	VC1715 ordered from CGC and backcrossed 4× to N2
MGH888	crn-3(ok2269); osIs158[eft-3p::AtTIR1(F79G)::mRuby] II; [GFP::AID*::3x-FLAG::fasn-1]	Constructed for this paper. MGH887 crossed into MGH770
IG274	*frIs7 [nlp-29p::GFP+col-12p::DsRed] IV*	Ordered from CGC
MGH823	*fasn-1(fr8)I; frIs7 [nlp-29p::GFP+col-12p::DsRed] IV*	IG348 ordered from CGC and backcrossed 8× to N2
*bud-23(5768)*	*C27F2.4 / bud-23(5768)*	Gifted by Greer Lab, Washington University St. Louis
COP262	*unc-119(ed3)III; knuSi221 [fib-1p::fib-1(genomic)::eGFP::fib-1 3′ UTR*	Ordered from CGC
QK52 (previously JM43)	*rde-1(ne219)V; xkIs99 [wrt-2p::rde-1::unc-54 3'UTR]*	Gifted by Ruvkun Lab, MGH
MGH167	*sid-1(qt9);alxIs6[VHA-6p::SID-1::SL2::GFP]*	
JMC237	*nrde-3(tor131[GFP::3xFLAG::nrde-3]) X*	Ordered from CGC
JV2	jrIs2 [rpl-17p::Grx1-roGFP2+unc-119(+)]	Ordered from CGC
MGH989	[GFP::AID*::3x-FLAG::fasn-1];osIs158[eft-3p::AtTIR1(F79G)::mRuby] II.; rsks-1(ok1255)	Constructed for this paper

### Alkaline hypochlorite synchronization of *C. elegans*

To synchronize worms for experiments, gravid animals were collected in M9 medium and centrifuged at 4,400 × *g* for 30 s. Worms were resuspended in 6 mL of 1.3% bleach, 250 mM NaOH and shaken vigorously for 1 min. This first bleach exposure was followed by centrifugation for 30 s and a wash step in M9. A second bleach exposure was performed for 1 min followed by 3 to 4 washes in M9 to remove residual bleach. Eggs were resuspended in 7 to 12 mL of M9 and left to rotate for 16 to 24 h at 20 °C, allowing for a synchronized L1 population to hatch.

### Lifespan analysis

Strains were grown on NGM plates seeded with OP50-1 bacteria. Gravid were washed off the plates in M9 buffer. Worms were synchronized via alkaline hypochlorite treatment and left on a rotator at 20 °C for 18 to 24 h. L1 worms were dropped onto NGM plates seeded with the appropriate bacteria as a food source and allowed to grow to the L4 stage. A total of 50 to 60 L4 worms were transferred to each experimental plate supplemented with 200 µM 5-fluorodeoxyuridine (FUdR) to suppress reproduction and progeny development. All lifespans were conducted at 20 °C and were post-developmentally treated with RNAi and drugs starting from L4 of development unless otherwise specified.

### Development analysis

RNAi plates were prepared as described above. Plates were treated with the appropriate drugs over the top of the bacterial lawn and dried in a laminar flow hood. Animals were synchronized via alkaline hypochlorite synchronization and L1 worms were dropped onto the appropriate experimental plates. Worms were subsequently grown at 20 °C. At the indicated time points of 72 and 120 h, animals were scored for their transition from larvae to adults by the appearance of the vulva. Drug treatments included phenformin, 5-PH-IAA, and cycloheximide. Phenformin hydrochloride powder was reconstituted in water to a concentration of 90 mM. Meanwhile 5-PH-IAA powder was reconstituted in DMSO at a final concentration of 100 mM, and cycloheximide powder was reconstituted in ethanol at a final concentration of 200 mM. These stock solutions were diluted as necessary to obtain the desired final concentration while accounting for the 10 mL of agar in the final volume. Drug treatments were added to the plates by applying 500 µL of drug solution over the top of the agar followed by drying in a laminar flow hood. Control plates contained equivalent quantities of DMSO and ethanol.

### 96-well metabolism RNAi library screen

96-well RNAi high-throughput microscopic assessment of animals was performed as previously described (Pino et al. 2013). Larval development of eft-3p::AtTIR1(F79G)::mRuby; 3xFLAG::AID*::GFP::FASN-1 animals was assessed following 72 and 120 h of combined 4.5 mM phenformin and 5 µM 5-PH-IAA treatment. Firstly, animals were bleach synchronized for 18 h, and approximately 50 to 75 L1s were subsequently dropped onto 96-well RNAi bacto agar plates containing 4.5 mM phenformin and 5 µM 5-PH-IAA and seeded with 1,046 individual RNAi clones corresponding to genes annotated as metabolism-related according to Gene Ontology terms. Animals were then incubated for at 20 °C prior to microscopic analysis. The number of viable adults were counted per well under dissecting microscope and compared to the percent reaching adulthood in EV and vehicle control wells. The top 91 RNAi clones with the greatest number of viable adults were selected for retesting on larger 60 mm × 15 mm petri dishes with an additional series of EV control plates to identify candidate targets. Visualization of the screen results were performed using GraphPad Prism 10.

### qRT-PCR analyses

qRT-PCR was performed as previously described (Cedillo et al. 2023). Approximately 1,000 synchronized animals of the stage and treatment conditions indicated in the figure legends were harvested using M9 buffer and washed an additional 3 times to remove residual bacteria. Animals were homogenized using a Qiagen TissueLyzer II with metal beads, centrifuged for 10 min at 12,000 × *g* to pellet cuticle and cellular debris, and RNA was extracted from the supernatant using RNAzol RT (Molecular Research Center) according to manufacturer instructions. RNA was treated with RNase-free DNAse prior to reverse transcription using the Quantititect Reverse Transcription kit (Qiagen). qRT-PCR was conducted using Quantitect SYBR Green PCR reagent (Qiagen) following manufacturer instructions on a Bio-Rad CFX96 Real-Time PCR system (Bio-Rad). Expression levels of tested genes were presented as normalized fold changes to the abundance control genes (as indicated in the figure legends) using the ΔΔCt method (Livak and Schmittgen 2001). The following primers were used for expression analysis:

*bud-23*, Forward: 5′ ATGAAATGGCTGAAAGAGCAC”
*bud-23*, Reverse: 5′ TACCGCAACCAATATCTAGCA 3′
*crn-3*, Forward: 5′ GAATGCACTCATGAACAAAGTC 3′
*crn-3*, Reverse: 5′ TAATGTTCGACTGATGAACCG 3′
eft-3, Forward: 5′ GTAAGGGATCTTTCAAGTACGC 3′ (housekeeping gene)
eft-3, Reverse: 5′ CATCGATGATGGTGATGTAGTAC 3′ (housekeeping gene)

### Puromycin incorporation assay

Puromycin incorporation was performed in *C. elegans* as previously described with some modifications (Clay et al. 2023). Overnight cultures of the appropriate RNAi clones were induced with IPTG for 2 h at a concentration of 175 µL of 1 M IPTG per 35 mL of bacterial culture for a final concentration of 5 mM IPTG. Bacterial cultures were then centrifuged and resuspended at a 10× concentration. Worms were incubated in a 1.5-mL microcentrifuge tube with 200 µL of 10× bacteria, 790 mL of S-buffer, and 10 µL of 50 mg/mL puromycin (final concentration 0.5 mg/mL) for 4 h at 20 °C on a rotator or rocker. Worms were then rinsed 3 times in PBS, flash-frozen in liquid nitrogen, and stored at −80 °C prior to processing for immunoblotting.

### Immunoblotting

Worm and cell lysates were prepared in RIPA buffer supplemented with protease and phosphatase inhibitors (Roche cOmplete, EDTA-free Protease Inhibitor Cocktail, sodium pyrophosphate, sodium fluoride, sodium orthovanadate, β-glycerophosphate, sodium molybdate, and okadaic acid). Samples were sonicated at 4 °C using a Q-sonica set at 40% amplitude at intervals of 30 s/30× on and off for a total of 20 min. Samples were then spun down for 15 min at 14,000 rpm at 4 °C to pellet debris, and the supernatant was transferred to a fresh tube. A bicinchoninic acid (BCA) assay was used to quantify the protein in the supernatant of each sample. The concentration of the samples was then normalized to the sample with the lowest concentration, using RIPA lysis buffer as a diluent. 4× Laemmli sample buffer (Bio-Rad) supplemented with 10% (v/v) β-mercaptoethanol was added to each sample for a final 1× concentration. Samples were boiled at 95 °C for 5 min and then stored at 20 °C until used for SDS-PAGE. Protein samples were loaded into 4% to 15% Mini-PROTEAN TGX Precast Protein Gels (Bio-Rad) and run at 150 volts for 1 to 1.5 h in a Bio-Rad Mini Protean Tetra Cell gel system. Bio-Rad's Precision Plus Dual Color standard was used as a protein ladder. Proteins were transferred to a nitrocellulose membrane via electrophoretic transfer using a wet tank-transfer system run at 100 V for 1 h at 4 °C in a 20% methanol transfer buffer. Ponceau staining was used to visualize proteins before blocking for 1 h in 5% milk dissolved in TBST. Primary antibody incubation was conducted overnight at 4 °C. The following antibodies were used: mouse monoclonal anti-Actin (C4) (Abcam, Cat# ab14128, 1:2,500), rabbit monoclonal anti-GFP (D5.1) (Cell Signaling Technology, Cat# 2956, 1:1,000), mouse monoclonal anti-puromycin (12D10) (Millipore Sigma, Cat# MABE343, 1:1,000), and mouse monoclonal anti-FLAG (M2) (Millipore Sigma, Cat# F3165, 1:2,500).

M were then washed 3 times with 1× TBST the next day. For primary antibody immunodetection, a 1:5,000 dilution of Goat anti-rabbit HRP conjugate or Goat anti-mouse HRP conjugate (GE Healthcare) in a 5% bovine serum albumin (BSA) 1× TBST solution was used, incubating membranes for 1 h rocking at room temperature prior to the addition of SuperSignal West-Pico chemiluminescent substrate (Thermo Pierce). Blots were visualized using an Invitrogen iBright Imaging system. To reprobe blots for the loading control, 10 mL of Thermo Fisher's Restore PLUS Western Blot Stripping Buffer was added to the membrane and left to rock at room temperature for 10 min before washing once in TBS and then re-blocking in 5% milk in TBST for 1 h. Blots could then be incubated in the desired primary antibody overnight as previously described. Western blots were quantified for band intensity using ImageJ software's rectangle selection tool. Visualization and post hoc statistical analysis of the results were performed using GraphPad Prism 10.

### Live worm imaging/quantification of fluorescence intensity

Worms were mounted onto a 2% agarose pad, sedated with 2.5 mM levamisole, and imaged using a Leica DM6 B microscope with Thunder Imaging capabilities under the appropriate bright-field or fluorescent channel and magnification. Images were quantified by manually tracing animals using ImageJ software's polygon selections tool. Visualization and post hoc statistical analysis of the results were performed using GraphPad Prism 10.

### Fixed nucleolar imaging in *C. elegans*

Staining for the imaging of nuclei and nucleoli was performed as previously described in Sharifi et al. (2024) with a few modifications. Worms were collected by washing plates and rinsed 3 times to remove residual bacteria. In a 1.5- mL microcentrifuge tube, worms were fixed in 1 mL of 50% ethanol (diluted in water) for 5 min and then washed in PBS 3 times. Worms were then incubated in 1 mL of 10 µM Hoechst 3342 for 15 min in the dark. Worms were mounted directly onto glass microscope slides and imaged at 63× magnification under oil immersion using a Leica DM6 B microscope. The nuclear stain Hoechst 3342 was imaged under a BFP channel while the nucleolar *fib-1:GFP* signal was imaged under a GFP channel. Images were quantified by manually tracing the nuclei and nucleoli using ImageJ software's polygon selections tool. At least 3 hypodermal cells per worm were imaged. Nucleolar size was calculated by dividing the nucleolar area of each cell by its nuclear area, thus normalizing to each individual cell. The average nucleolar to nuclear ratio for each worm was then calculated. Visualization and post hoc statistical analysis of the results were performed using GraphPad Prism 10.

### RNA-sequencing analyses

For bulk RNA-sequencing analyses, 1,000 worms of the indicated strain, genotype, or treatment per sample and replicate were harvested using M9, washed 3 times to remove bacteria, and worm pellets were resuspended into 500 μL of RNAzol RT. Total RNA was subsequently extracted, and the genomic DNA was removed using the Direct-Zol RNA Miniprep Plus Kit (Zymo Research) following manufacturer's instructions. Total extracted RNA was evaluated for quality control using a NanoDrop One C Microvolume UV-Vis Spectrophotometer (Thermo Fisher). Samples were sent to Azenta (Genewiz) for additional quality control, library preparation, and mRNA sequencing. Samples were validated for RNA integrity with a RIN score >9 and DV200 >70 using an RNA TapeStation 4200 (Agilent). Illumina library preparation was performed using polyA selection for mRNA species. Approximately 20 million paired-end 150-bp reads were generated per sample, with ≥80% of bases passing a Phred quality score ≥Q30. Fastq read quality control, adapter trimming, quality score filtering, and quasi-alignment were all performed using custom UNIX/Bash shell scripts on the Mass General Brigham ERISTwo Scientific Computing CentOS 7 Linux Cluster. Reads were analyzed for quality control using FastQC v0.11.8 (http://www.bioinformatics.babraham.ac.uk/projects/fastqc) and MultiQC v1.19^81,82^. Reads were then filtered for Illumina adapter contamination, truncated short reads, or low-quality base calls using BBDuk (BBTools). The subsequently trimmed and cleaned reads were then quasi-aligned to the *Caenorhabditis elegans* reference transcriptome annotation (WBcel235, Ensembl Release 105) using Salmon v1.9.0, correcting for GC content and sequencing bias using the command parameters “--gcBias” and “--seqBias” (Patro et al. 2017). All statistical analyses and visualizations were generated using the R v4.3.2 (www.r-project.org) Bioconductor v3.18 statistical environment on a local machine through Jupyter Notebook v6.4.10 (https://jupyter.org) (Huber et al. 2015). Quasi-aligned transcript quantification files for each sample were collapsed into gene-level count matrices using the R package tximport v1.30.0 (Soneson et al. 2015). Paired differential expression was calculated using R package DESeq2 v1.42.1 with a design formula of “∼ RNAi_Treatment” (Love et al. 2014). Genes were considered differentially expressed with a Benjamini–Hochberg false discovery rate (FDR) corrected *P-*value < 0.05 and an absolute log_2_-transformed fold change of 1 (Benjamini and Hochberg 1995). Comparison of differentially expressed genes with prior *C. elegans* mutant datasets was performed using WormExp v2.0 (Yang et al. 2016). Tissue expression prediction analysis of differentially expressed genes as indicated in the figure legends was performed using the Worm Tissue Prediction Server and data plotted using Graphpad Prism 10 (Kaletsky et al. 2018). Visualizations were post-edited for font, sizing, and appearance using Adobe Illustrator and the Adobe Creative Cloud Suite. All RNA-sequencing data have been uploaded to the NCBI Gene Expression Omnibus (GEO) under accession number GSE277946.

### NADPH/NADP+ and NADH/NAD+ ratiometric profiling

Ratiometric redox profiling was performed using the NADP/NADPH Ratio Red Fluorescence Assay Kit (eEnzyme, Cat# CA-N426) and the NAD/NADH Colorimetric Assay Kit (abcam, Cat# ab65348) according to manufacturer's instructions. To prepare lysates for reducing equivalent extraction, ∼1,000 nematode lysates for each condition and sample indicated were collected into M9 buffer supplemented with EDTA-free protease inhibitor tablets (Roche) and a phosphatase inhibitor cocktail containing sodium fluoride, sodium pyrophosphate, sodium orthovanadate, β-glycerophosphate, and okadaic acid. Samples were then sonicated at 40% amplitude in a QSonica Q800R3 water bath sonicator at 30 s ON/30 s OFF intervals for a total of 20 min at 4 °C, ensuring cuticle breakdown uniformly across each sample via dissecting microscope. Lysates were cleared by centrifugation at 21,000 × *g* at 4 °C for 10 min, and supernatants were retained in clean microcentrifuge tubes for NADPH/NADP+/NADH/NAD+ extraction. Fluorescence emission spectra were collected using a Spectramax i3x plate reader, and colorimetric measurements were collected using a Spectramax PLUS plate reader. Visualization and post hoc statistical analysis of the results were performed using GraphPad Prism 10.

### Statistical analysis

To determine statistical significance for developmental assays and quantification of fluorescence or western blot band intensity, GraphPad's Prism 10 software was used to run 1-way and 2-way ANOVAs with interaction testing. Unless otherwise noted, all ANOVAs were run with Sidak correction for multiple comparison testing and any comparisons shown in figures report the corrected *P*-values where *P* < 0.05 was the threshold for significance. Asterisks denote corresponding statistical significance: *P < 0.05; **P < 0.01; ***P < 0.001; and ****P < 0.0001.

For lifespan analysis experiments, the software OASIS 2 was used to determine statistical significance between the mean lifespans of different experimental conditions using Kaplan–Meier estimation and log-rank testing. OASIS 2 was also used to perform Cox proportional hazard testing. Unless otherwise specified, comparisons shown in figures and tables report the Bonferroni-corrected *P*-value where values where *P <* 0.05 was the threshold for significance. Asterisks denote corresponding statistical significance: *P < 0.05; **P < 0.01; ***P < 0.001; and ****P < 0.0001.

## Results

### RNAi knockdown of *crn-3* and *bud-23* protects against toxic reductive stress

As established in our previous studies, *fasn-1* is essential for protection against phenformin toxicity as it consumes excess reducing equivalents to prevent reductive death (Ahsan et al. 2026). However, it remains unclear how organisms might mitigate reductive stress in the absence of *fasn-1*, either by activating downstream genes or through parallel pathways. Combining RNA interference (RNAi) and auxin-inducible degron (AID) technology with pharmacological interventions, we harnessed the power of reverse genetics to identify genes whose knockdown mitigated the toxicity of a highly reductive cellular environment. To spatiotemporally visualize and knockdown *fasn-1*, we crossed animals bearing a somatically expressed *Arabidopsis thaliana* auxin receptor transport inhibitor response 1 protein transgene [AtTIR-1(F79G)] with animals expressing an endogenously tagged *N*-terminal 3xFLAG::AID*::GFP transgene (Zhang et al. 2015; Ahsan et al. 2026). This AID system was activated upon the administration of 5-phenyl-1H-indole-3-acetic acid (5-Ph-IAA) to effectively deplete FASN-1 protein levels.

Leveraging this AID system in tandem with RNAi technology, we sought to uncover epistatic genetic interactions that underlie this synergistic reductive death response. We performed a high-throughput reverse genetic screen, treating L1-stage *fasn-1*-deficient nematodes with phenformin and concomitant feeding of an established RNAi library targeting 1,046 metabolism associated genes (Fig. 1a). From this initial screen, we identified 2 genes—*crn-*3/CeExosc-10 and *c27f2.4/*CeBUD*-23*—whose loss significantly improved survival into adulthood (Fig. 1b). Specifically, *crn-3* is part of the RNA exosome, which is involved in apoptotic DNA degradation and the processing of the 5.8S and 18S rRNA (Tafforeau et al. 2013; Zhu et al. 2018). Meanwhile, *bud-23* is an rRNA methyltransferase that aids in the processing of 18s rRNA (Zhu et al. 2018).

**Fig. 1.**
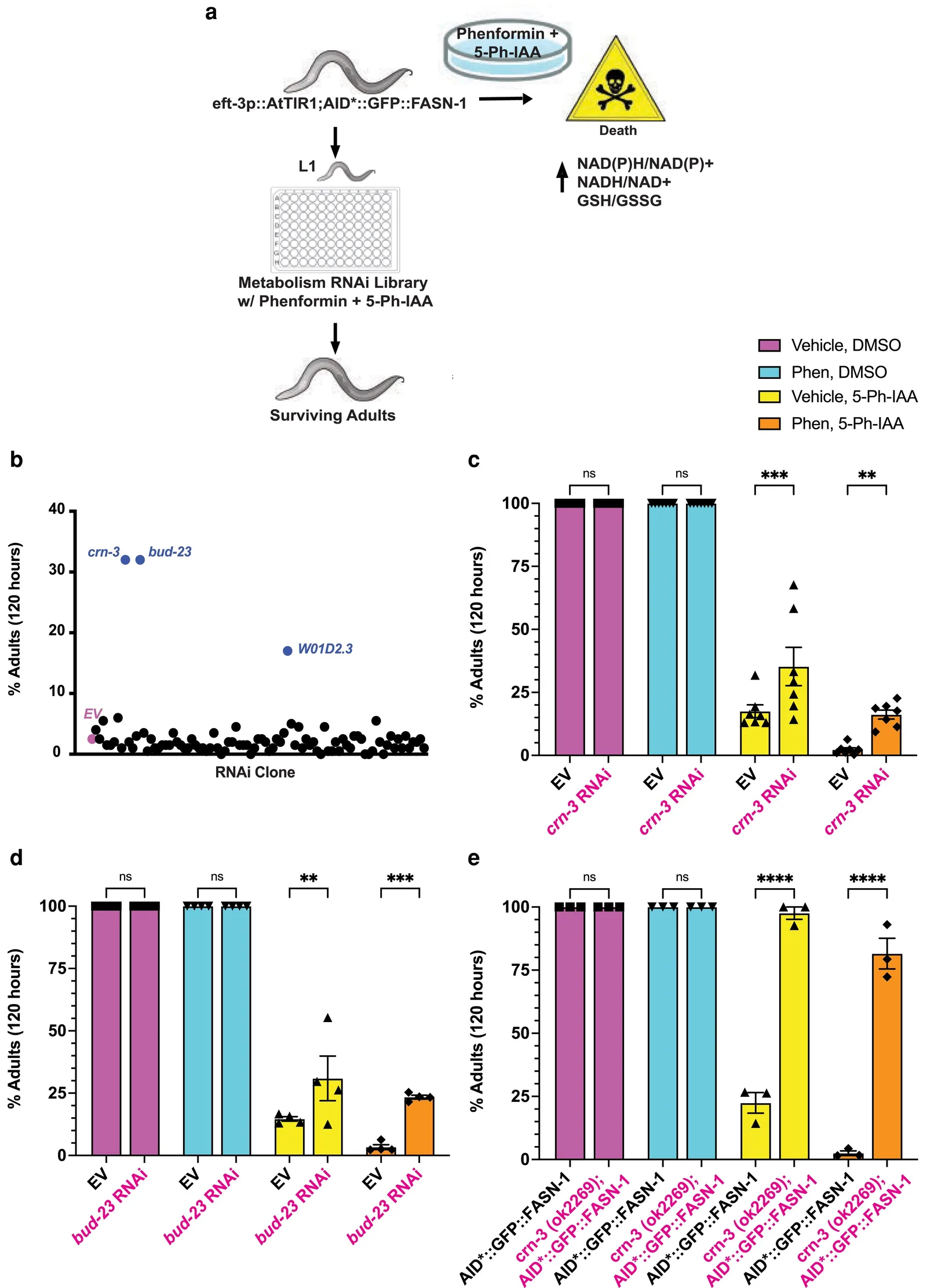
Reverse genetic screen identifies *crn-3* and *bud-23* RNAi as suppressors of biguanide-induced toxicity in the absence of *de novo fatty acid synthesis*. a) Design of a reverse genetic RNAi screen targeting metabolism genes. b) Secondary retesting of 91 RNAi clones of interest from initial screen. c to e) Quantification of developmental assays on (c) EV vs *crn-3* RNAi, (d) EV vs *bud-23* RNAi, and (e) control eft-3p::AtTIR1;AID*::GFP::FASN-1 animals vs crn-3(ok226); eft-3p::AtTIR1;AID*::GFP::FASN-1 mutants. Animals were treated with or without 5 µM 5-Ph-IAA and 4.5 mM phenformin from L1 of hatching. Experiments were performed in ≥3 biological replicates with technical triplicates. All error bars represent mean ± SEM unless otherwise noted. Interaction and significance were assessed in (c to e) using a 2-way ANOVA with Sidak correction of multiple comparison testing (see Supplementary Data Tables). Corrected *P*-values are shown in this figure. ^ns^*P* > 0.05, *P < 0.05, **P < 0.01, ***P < 0.001, ****P < 0.0001.

When animals were treated with 5-PhIAA from L1 of hatching, survival was significantly reduced with only 20% reaching adulthood after 120 h (Fig. 1c). When 5-Ph-IAA treatment was combined with a normally lifespan-extending dose of 4.5 mM phenformin from L1 of hatching, less than 5% of animals reaching adulthood (Fig. 1c). These results were consistent with the reductive death phenotype observed upon combining *fasn-1* RNAi with phenformin and other NADPH-promoting interventions such as malic acid and glucose supplementation (Ahsan et al. 2026).

We validated the RNAi knockdown efficiency for *crn-3* and *bud-23* in the animals via qRT-PCR (Supplementary Fig. 1a). RNAi of these genes resulted in the improved organismal survival of *fasn-1-*deficient worms with or without phenformin (Fig. 1, c and d; Supplementary Fig. 1b). To corroborate our findings genetically, we crossed AID*::GFP:: FASN-1 animals with a *crn-3(ok2269)* null mutant, which has a 1,049-bp deletion within the coding sequence. We observed that *crn-3* mutant worms exhibited delayed development under control conditions, taking approximately 24 h longer to reach the L4 stage compared to wild-type animals, which is consistent with impaired ribosome biogenesis (Supplementary Fig. 1, c and d). Despite this significant delay in development, approximately 100% of *crn-3* mutants reached adulthood after 120 h of 5-Ph-IAA treatment (Fig. 1e; Supplementary Fig. 1d). A similar rescue of reductive death was observed in *crn-3* mutants treated with both 5-Ph-IAA and phenformin, where 75% reached adulthood after 120 h (Fig. 1e).

To rule out a loss of AID knockdown efficiency in *crn-3* mutants as a potential confounder of results, we evaluated FASN-1 expression upon 5-Ph-IAA treatment. Immunoblotting revealed that *crn-3* mutant animals basally overexpress FASN-1 protein compared to the wild-type animals, implicating *crn-3* as a possible negative regulator of *fasn-1* (Supplementary Fig. 1, e and f; See Supplementary Immunoblots). Importantly, the immunoblot also confirmed that genetic ablation of *crn-3* did not cause leaky AID-knockdown efficacy, given that FASN-1 levels were equally depleted in wild-type and mutant animals (Supplementary Fig. 1, e and f; See Supplementary Immunoblots).

### Genetic loss of *crn-3* protects against accelerated death associated with post-developmental phenformin and *fasn-1* RNAi treatment

To determine if *crn-3* and *bud-23* deficiency was sufficient to protect against reductive stress later in the worm's lifespan, we performed longitudinal survival analyses where we treated animals with phenformin with or without *fasn-1* RNAi starting at L4 of hatching. We observed that *crn-3* null mutants were slightly short-lived compared to wild-type animals and still exhibited phenformin-mediated lifespan extension (Fig. 2, a to c). Consistent with our previous work, both wild-type and *crn-3* mutant animals treated with phenformin and *fasn-1* RNAi exhibited a synthetic lethal phenotype (Fig. 2, a to c). However, *crn-3* mutants experienced a significant delay in the onset of reductive death by about 2 d (Fig. 2, a to c). Contrastingly, *bud-23* mutant animals did not display a delay in the onset of reductive death (Supplementary Fig. 2, a to c). Both *crn-3* and *bud-23* mutants exhibited blunted phenformin-mediated lifespan extension compared to wild-type (Fig. 2, a to c; Supplementary Fig. 2, a to c), suggesting that the function of these 2 genes may be partially required for phenformin-mediated lifespan extension, but are dispensable under acute phenformin-induced stress.

**Fig. 2.**
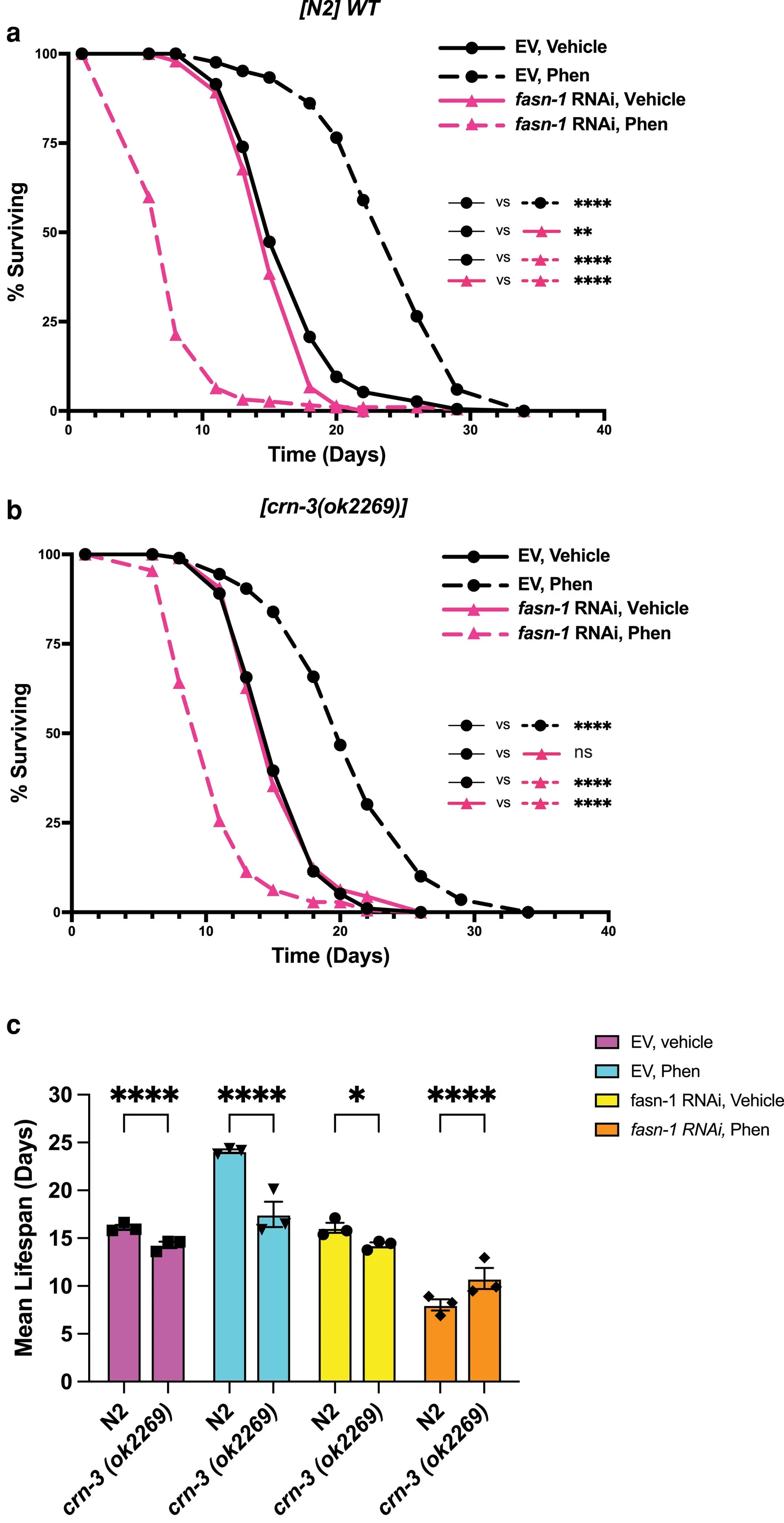
Genetic loss of *crn-3* delays reductive death in phenformin-treated, *fasn-1-*deficient animals. a and b) Lifespan analyses of (a) wild-type and (b) *crn-3(ok2269)* animals fed EV or *fasn-1* RNAi and treated with vehicle or 4.5 mM phenformin from L4 of hatching. c) Visualization of mean lifespan for each condition in (a and b). Three independent biological replicates were performed in technical triplicate. All mutant animals were backcrossed 4 to 6 times to wild-type animals to remove background mutations. Significance was assessed in (a and b) using log rank analyses and a cox proportional hazard regression. Significance was assessed in (c) using a 2-way ANOVA with interaction analysis and sidak correction for multiple comparisons (see Supplementary Data Tables). ^ns^*P* > 0.05, *P < 0.05, **P < 0.01, ***P < 0.001, ****P < 0.0001.

### Hypodermal *fasn-1* expression is protective against phenformin toxicity

We sought to determine which tissues required *fasn-1* expression to prevent the phenformin toxicity. Interestingly, RNA sequencing and enrichment analysis revealed that intestinal, hypodermal, and epithelial genes were most differentially expressed when comparing phenformin alone and combined *fasn-1* RNAi and phenformin treatment (Fig. 3a). Using tissue-specific RNAi strains, we performed lifespan analyses. Notably, hypodermal but not intestinal *fasn-1* RNAi knockdown phenocopied the accelerated demise observed in WT animals (Fig. 3, b to d). This suggests that the *fasn-1* expression in the hypodermis could play an especially important role in the defense against reductive stress.

**Fig. 3.**
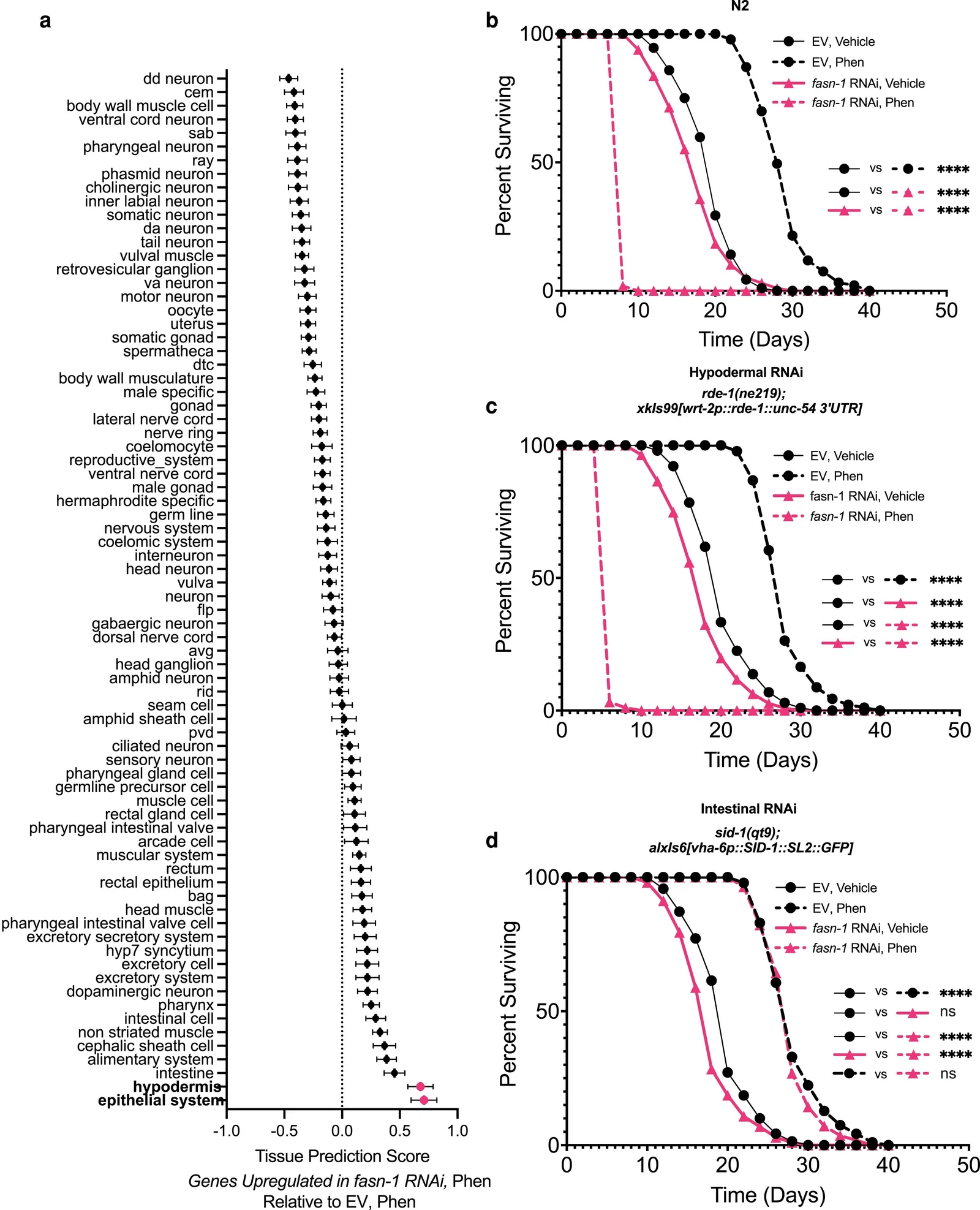
Hypodermal expression of *fasn-*1 protects against reductive death. a) Tissue prediction analysis of genes differentially upregulated by *fasn-1* RNAi with 4.5 mM phenformin relative to EV with 4.5 mM phenformin. Genes were selected based on a log_2_ fold change greater than 1 and an adjusted *P-*value < 0.05. Tissue prediction scores were generated for the selected list using https://worm.princeton.edu. b to d) Lifespan analysis of (b) N2 wild-type (c) hypodermal specific RNAi competent animals (d) intestinal specific RNAi competent animals treated with or without *fasn-1* RNAi and 4.5 mM phenformin from L4 of hatching. a to d) Results are representative of 3 biological replicates. Significance was assessed for (a) using DESeq2 Wald testing with Benjamini–Hochberg FDR correction, and (b to d) using log rank analyses alongside cox proportional hazard regression (see Supplementary Data Tables). ^ns^*P* > 0.05, *P < 0.05, **P < 0.01, ***P < 0.001, ****P < 0.0001.

### Combined *fasn-1* deficiency and phenformin treatment alters nucleolar size, translational efficiency, and the ER stress response

The observation that the knockdown of *crn-3* and *bud-23*—2 genes involved in rRNA biogenesis—significantly improved survival prompted an investigation into the effects of phenformin-induced reductive stress on rRNA synthesis. We hypothesized that an overexpression of rRNA could be a pathological consequence of reductive death. To test this, we performed experiments to assess nuclear morphology using a translational reporter for the nucleolar protein fibrillarin (ie *fib-1*) while co-staining for nuclear DNA (Fig. 4a). Nucleolar size can be an indicator of nucleolar activity. Enhanced rDNA transcription, carcinogenesis, and shortened lifespan are positively correlated with larger nucleoli, whilst small nucleoli are reciprocally predictive of pro-longevity response (Ma et al. 2016; Tiku et al. 2017).

**Fig. 4.**
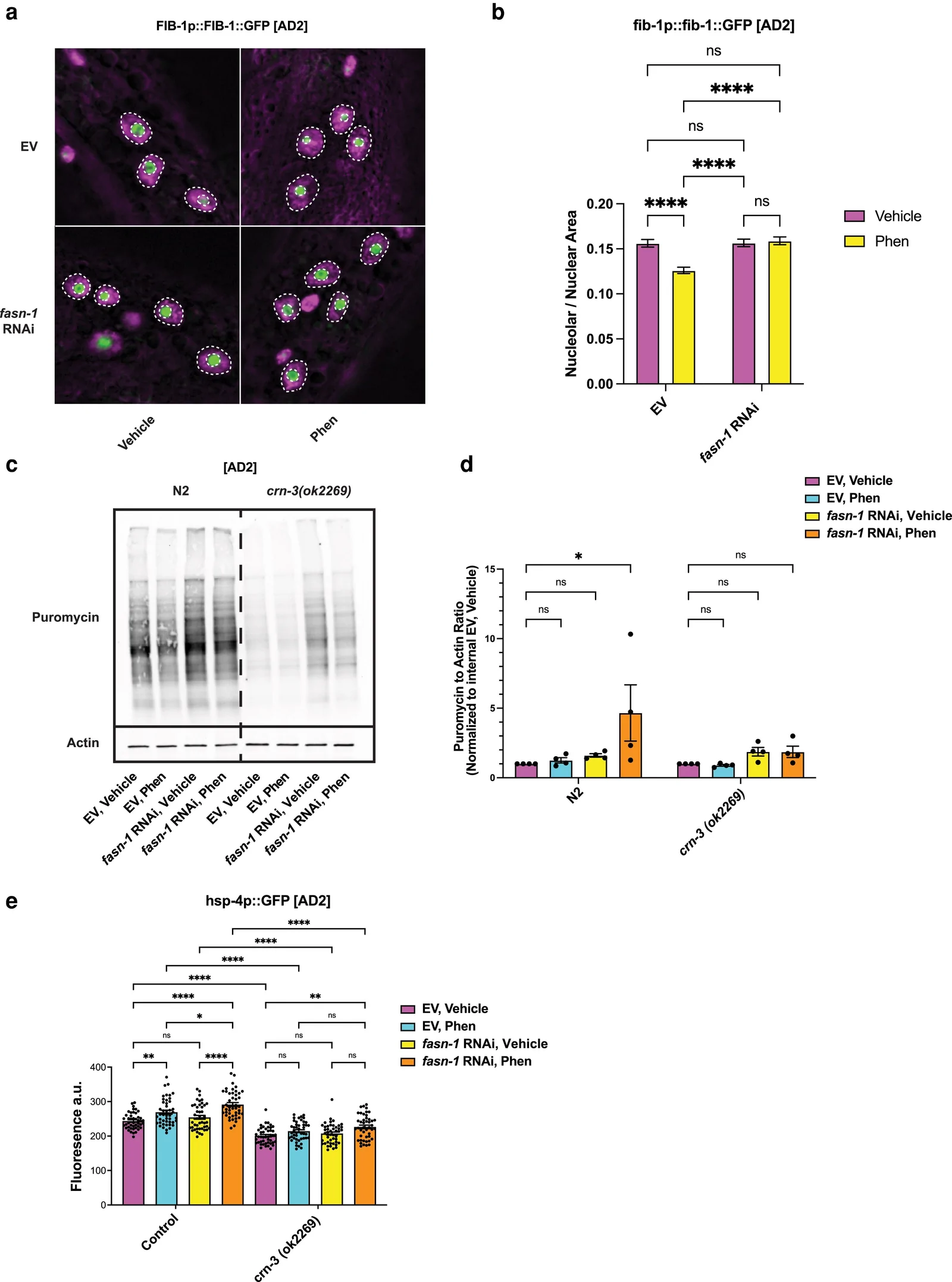
*fasn-1* is necessary for phenformin-mediated reduction of nucleolar size. a) Representative images of hypodermal cells. Cells were imaged at 63× magnification under a GFP filter to detect *FIB-1::GFP* expression. Worms were additionally stained with Hoechst 33342 to visualize nuclear DNA and imaged under a BFP filter. Fluorescent images have been overlayed and nuclei and nucleoli have been outlined. b) Quantification of nucleolar/nuclear area ratio in animals treated with or without 4.5 mM phenformin and *fasn-1* RNAi from L4 to AD2. Data are compiled from 3 independent biological replicates with a total of 60 worms imaged per condition. c) Representative SUrface Sensing of Translation (SUnSET) puromycin incorporation immunoblotting in wild-type and *crn-3(ok2269)* animals treated with or without 4.5 mM phenformin and *fasn-1* RNAi from L4 to AD2. d) Quantification of puromycin incorporation immunoblots. Experiment was performed in 4 biological replicates with approximately 1,000 animals collected per condition. Due to the developmental delay in *crn-3(ok2269)* mutants, puromycin incorporation assays were performed a day after the wild-type control. e) Quantification of fluorescence in worms expressing a *hsp-4p::GFP* transcriptional reporter in wild-type and *crn-3(ok2269)* animals. Data is inclusive of 3 biological replicates with a total of 45 worms imaged per condition. All error bars represent mean ± SEM unless otherwise noted. b, d, and e) Significance was assessed using 2-way ANOVA with interaction analysis and Sidak correction of multiple comparison testing (see Supplementary Data Tables). Corrected *P*-values are shown in figure. ^ns^*P* > 0.05, *P < 0.05, **P < 0.01, ***P < 0.001, ****P < 0.0001.

Inhibition of hypodermal nucleolar activity and ribosome biogenesis have been implicated in pro-longevity responses and stress resistance (Tiku, et al. 2017; Dalton and Curran 2018; Zhao et al. 2023). And as previously mentioned, we identified the hypodermis as the critical site of action for *fasn-1-*mediated protection against phenformin toxicity. Therefore, we focused on the nucleolar morphology of hypodermal cells. We observed that a lifespan-extending dose of 4.5 mM phenformin significantly reduced the nucleolar-to-nuclear area ratio in hypodermal cells imaged in the tail region. Notably, this effect was totally reversed upon RNAi knockdown of *fasn-1*, suggesting that *fasn-1* may act as a negative regulator of rRNA synthesis in the hypodermis (Fig. 4b).

We wondered whether enhancing nucleolar activity under combined *fasn-1* RNAi and phenformin treatment might correlate with an aberrant increase in downstream translation and whether *crn-3* might be inhibiting translation to prevent demise. Puromycin incorporation assays revealed that *fasn-1* RNAi may enhance nascent protein synthesis in both wild-type and *crn-3* mutant animals, although it was not statistically significant (Fig. 4, c and d; see Supplementary Immunoblots). Notably, the combination of *fasn-1* RNAi and phenformin significantly increased translation in wild-type animals but not *crn-3* mutants (Fig. 4d; see Supplementary Immunoblots). However, post hoc analysis did not find a significant interaction between the strain and treatment condition, indicating that the overall effect on translation across conditions is the same for both strains (see Supplementary Data Tables).

Given the observation that *fasn-1* RNAi combined with phenformin treatment may augment rRNA expression and therefore downstream translation, we wondered whether broader inhibition of translation could rescue lethality. We leveraged both pharmaceutical and genetic methods to inhibit translation. Cycloheximide is an antibiotic that acts by inhibiting translation elongation and has been shown to be effective in *C. elegans* at nonlethal concentrations between 100 µM and 200 µM (Clay et al. 2023; Zhao et al., 2026). Notably, cycloheximide at these concentrations did not significantly rescue the synthetic lethal effect observed upon combined AID knockdown of *fasn-1* and phenformin treatment (Supplementary Fig. 3a). We also targeted translation initiation via *rsks-1*—an ortholog of human ribosomal protein 6 (RPS6). Loss-of-function *rsks-1(ok1255)* mutants—which contain a 1.6-kilobase deletion of the gene—also failed to significantly rescue death (Supplementary Fig. 3b). Together, these results could suggest that inhibition of *crn-3* may protect against reductive stress through pathways unrelated to possible inhibitory effects on downstream global translation. Instead, inhibition of rRNA synthesis may be rewiring downstream signaling pathways to protect against stress.

Translation control is essential for maintaining proteostasis. Additionally, chronic reductive stress can disrupt the pro-oxidative protein folding environment in the ER, leading to pathological outcomes (Sunny et al. 2025). We wondered whether this aberrant increase in translation could be overwhelming the endoplasmic reticulum's protein-folding capacity, potentially causing proteotoxic effects. Using a strain expressing a transcriptional reporter for the heat shock protein *hsp-4*, we observed that phenformin treatment alone activated the UPR^ER^ stress response (Fig. 4e). This is consistent with existing literature showing that metformin can differentially activate ER stress signaling pathways without triggering apoptosis (Quentin et al. 2012). Notably, when phenformin was combined with *fasn-1* RNAi, a mild synergistic activation of the ER stress response occurred, which corresponded with reductive death (Fig. 4e). This could suggest that loss of *fasn-1* rewires the ER stress pathways that are activated upon phenformin treatment.

We wondered whether the *crn-3* inhibition might protect against ER stress. Interestingly, *hsp-4* expression was decreased across conditions, and phenformin failed to induce *hsp-4* expression in *crn-3* mutants but still had a mild synergistic induction when combined with *fasn-1* RNAi (Fig. 4e). However, we did not find significant interaction between the variables of strain and treatment condition. This suggests that *crn-3* is a positive regulator of *hsp-4*, but also that *crn-3* mutants are still susceptible to ER stress caused by *fasn-1* RNAi and phenformin treatment.

### Enhanced rRNA biogenesis exacerbates phenformin-induced hypodermal stress response in the *fasn-1-*deficient animals

As previously suggested, increased expression of hypodermal genes occurs upon tandem phenformin and *fasn-1* RNAi treatment (Fig. 3a). Knockdown of *fasn-1* has been shown to trigger the induction of hypodermal stress response gene *nlp-29* (Lee et al. 2010). RNA sequencing revealed that phenformin and *fasn-1* RNAi synergize to drive elevated *nlp-29* expression (Fig. 5a). We verified these results at the protein level by treating an integrated *nlp-29p:GFP;col-12p::dsRed* translational reporter strain with *fasn-1* RNAi and phenformin. Notably, the expression of *nlp-29* was temporally associated with the mean time point of death for each condition in the lifespan assays (Fig. 5b). Given our earlier results suggesting that *fasn-1* may be a negative regulator of nucleolar size, we wondered whether aberrant overexpression of rRNAs could be driving the hypodermal stress observed in *fasn-1-*deficient animals treated with phenformin. Inhibition of *ncl-1,* a histone chaperone protein and known suppressor of nucleolar size and rRNA biogenesis, has been associated with accelerated aging in *C. elegans* (Tiku et al. 2017). Interestingly, we observed a slight yet significant activation of the *nlp-29* reporter when *ncl-1* RNAi was combined with phenformin treatment (Fig. 5, c and d). However, analysis did not find any interaction between RNAi and drug treatment, suggesting that just enhancing rRNA expression may not significantly change the effect of phenformin on the hypodermal stress response.

**Fig. 5.**
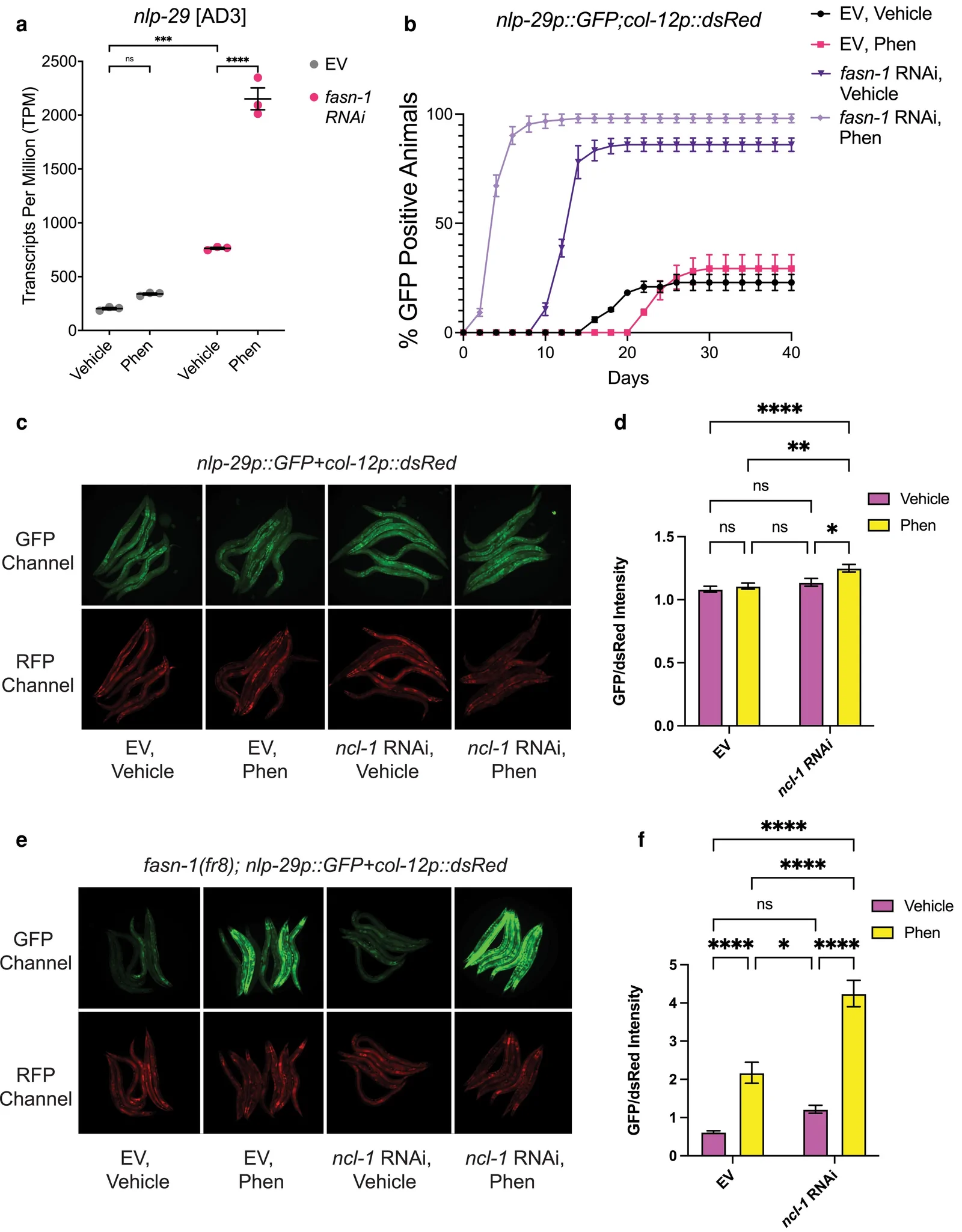
*ncl-1* RNAi synergistically activates hypodermal stress response gene *nlp-29* in combination with phenformin of *fasn-1* knockdown. a) Abundance of *nlp-29* mRNA transcripts in wild-type animals treated with or without *fasn-1* RNAi and 4.5 mM phenformin from L4 of hatching to AD3. b) Percentage of GFP-positive *nlp-29p::GFP;col-12p::dsRed* animals over time when treated with or without *fasn-1* RNAi and 4.5 mM phenformin from L4 of hatching. c and d) Representative images and quantification of fluorescence in *nlp-29p::GFP;col-12p::dsRed* animals treated with or without *ncl-1* RNAi and 4.5 mM phenformin from L4 of hatching to AD2. e and f) Representative images and quantification of fluorescence of *fasn-1(fr8); nlp-29p::GFP;col-12p::dsRed* animals treated with or without *ncl-1* RNAi and 4.5 mM phenformin from L4 of hatching to AD2. For (a, b, d, and f) experiments were performed in 3 biological replicates. c and e) worms were imaged at 5× magnification under green and red fluorescent channels. In (d and f) a total of 150 worms were imaged per condition and fluorescent intensity was quantified using ImageJ. All error bars represent mean ± SEM unless otherwise noted. Significance was assessed in (a, d, and f) using a 2-way ANOVA with interaction analysis and Sidak correction of multiple comparison testing (see Supplementary Data Tables). Corrected *P*-values are shown in figure. ^ns^*P* > 0.05, *P < 0.05, **P < 0.01, ***P < 0.001, ****P < 0.0001.

We wondered whether enhancing rRNA expression in tandem with *fasn-1* inhibition might exacerbate this hypodermal stress response. We subsequently crossed the *nlp-*29 reporter into a *fasn-1(fr8)* hypomorphic strain. Consistent with the RNA sequencing results, *fasn-1(fr8)* animals exhibited a synergistic activation of *nlp-29* when treated with phenformin (Fig. 5, e and f). Combined, these data suggest that aggravated hypodermal induction of *nlp-29* expression may act as a biomarker of reductive death where biguanide treatment synergistically enhances the primed epidermal stress state in *fasn-1-*deficient animals. We also discovered that combining both *fasn-1* and *ncl-1* deficiency with phenformin resulted in synergistic activation of *nlp-29* to a degree that exceeded all other experimental groups (Fig. 5, e and f). These findings suggest that enhancing rRNA biogenesis may increase sensitivity to reductive stress caused by biguanides and *fasn-1* deficiency.

### Inhibition of rRNA transcription, modification, and processing protects against reductive stress

Given that the canonical functions of *crn-3* and *bud-23* converge upon rRNA biogenesis and that enhancing rRNA transcription hyper-sensitized animals to hypodermal stress caused by combined phenformin and *fasn-1* deficiency, we speculated that inhibiting rRNA biosynthesis could prevent reductive death. The production of functional rRNA is a highly complex process involving many different posttranscriptional modifications and translocation from the nucleolus to the cytoplasm (Dörner et al. 2023) (Fig. 6a). We were interested to see whether impairing various steps in RNA polymerase I-mediated rRNA synthesis could improve survival outcomes under reductive stress conditions during development.

**Fig. 6.**
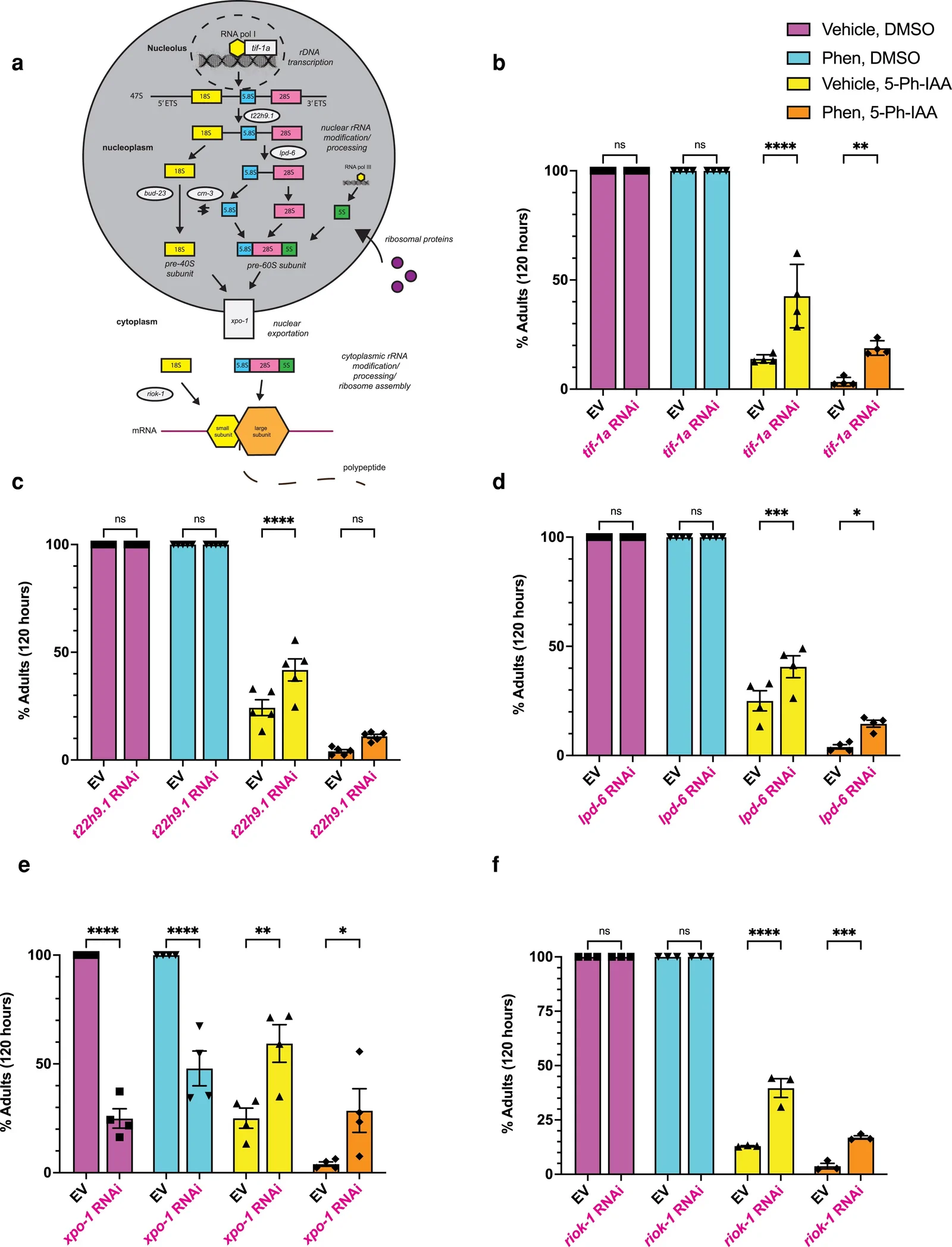
Inhibition of rRNA biogenesis significantly reverses reductive death. a) Simplified schematic of RNA polymerase I-mediated rRNA synthesis pathway. RNA polymerase I binds to ribosomal DNA in the nucleolus and transcribes the tri-cistronic 47S pre-rRNA transcript. Subsequent cleavage, modification, and exportation of the rRNA from the nucleus into the cytoplasm for final processing occurs to produce mature 18S rRNA, 5.8S rRNA, and 28S rRNA. Together with ribosomal proteins and 5S rRNA—which is transcribed separately by RNA polymerase III—rRNAs are assembled to form the small and large ribosomal subunits. b to f) Quantification of development in eft-3p::AtTIR1;AID*::GFP::FASN-1 animals was fed RNAi targeting genes involved in rRNA biogenesis and treated with or without 5 µM 5-Ph-IAA and 4.5 mM phenformin from L1 of hatching. Experiments were performed in >3 biological replicates in technical triplicate. All error bars represent mean ± SEM unless otherwise noted. Significance was assessed in (d, e, and h) using a 2-way ANOVA with interaction analysis and Sidak correction of multiple comparison testing (see Supplementary Data Tables). Corrected *P*-values are shown in figure. ^ns^*P* > 0.05, *P < 0.05, **P < 0.01, ***P < 0.001, ****P < 0.0001.

We inhibited rRNA synthesis at its source by targeting RNA polymerase I transcription initiation factor *tif-1a/*CeRRN-3. The results of our developmental assays revealed that *tif-1a* RNAi significantly increased the number of surviving *fasn-1-*deficient animals treated with and without phenformin from L1 of hatching (Fig. 6b; Supplementary Fig. 4). Similar results were obtained upon RNAi knockdown of nuclear rRNA processing genes *t22h9.1/*CeRRP*-36* and *lpd-6/*PPAN-P2RY11/CePPAN-P2RY11 (Fig. 6, c and d; Supplementary Fig. 4). RNAi knockdown of *xpo-1*—the exportin that facilitates the translocation of rRNA transcripts and ribosomal subunits from the nucleus into the cytosol for downstream ribosome assembly—also resulted in a significant resistance to reductive stress despite *xpo-1* RNAi itself disrupting development (Dörner et al. 2023) (Fig. 6e; Supplementary Fig. 4). Finally, RNAi knockdown of *riok-1*/CeRIO1*—*which facilitates the maturation of the 18S rRNA in the cytoplasm—also significantly reversed reductive death (Dörner et al. 2023) (Fig. 6f; Supplementary Fig. 4). To confirm that the presence of dsRNA alone does not explain this phenomenon, we used a nontargeting RNAi clone expressing dsRNA homologous to the hygromycin resistance gene (hygR), which is not expressed in *C. elegans*. This nontargeting RNAi clone failed to rescue death in AID*::GFP::FASN-1 animals treated with the auxin ortholog and phenformin (Supplementary Fig. 4b). These results suggest that specifically disrupting rRNA at various points during ribosome biogenesis—from early-stage transcription to nuclear processing, to nuclear exportation, to late-stage cytoplasmic maturation—can activate a nucleolar stress response that protects against toxic reductive stress.

### Inhibiting *crn-3* increases tolerance of NADPH and NADH reductive stress and decreases reduced glutathione levels

Previous work done by our lab suggests that the accumulation of reducing equivalents is the likely cause of accelerated death upon combined phenformin and *fasn-1* RNAi (Ahsan et al. 2026). This led us to investigate whether inhibiting rRNA biogenesis prevents the buildup of reducing equivalents to improve survival outcomes. To answer this question, we conducted ex vivo and in vivo ratiometric fluorescence assays to investigate redox dynamics in wild-type vs *crn-3*(ok2269) mutants. Interestingly, NADPH/NADP+ and NADH/NAD+ ratios were significantly higher in *crn-3* mutants across all conditions compared to wild-type animals (Fig. 7, a and b). However, we were also interested in determining whether *crn-3* mutants respond differently than wild-type animals when challenged by reductive stressors. Thus, we looked closer at the trends within each strain.

**Fig. 7.**
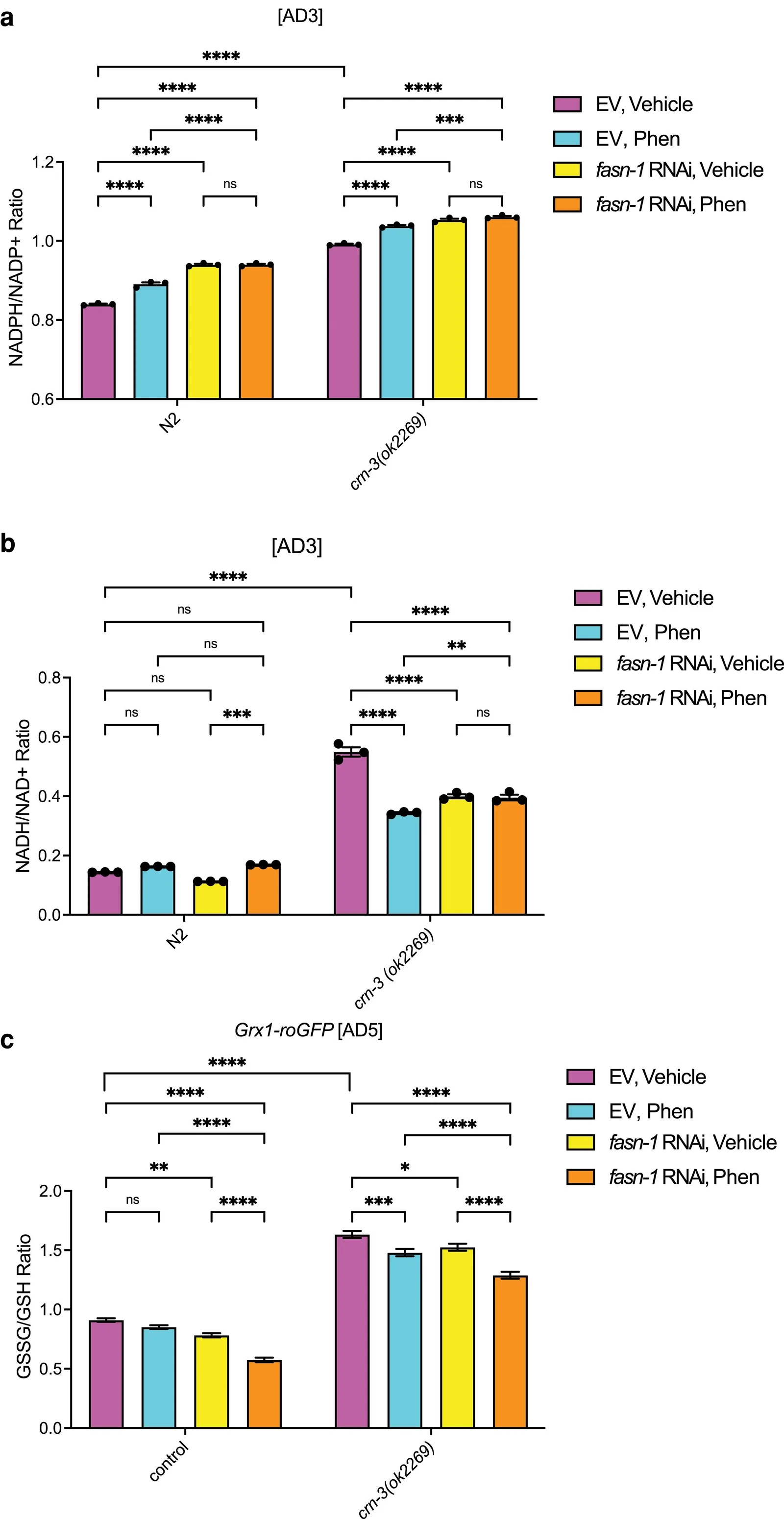
Inhibition of *crn-3* via genetic mutation alters NADPH/NADP+, NADH/NAD+, and GSSG/GSH ratios. a and b) Quantification of the NADPH/NADP+ (a) and NADH/NAD+ (b) ratios in N2 wild-type versus *crn-3(ok2269)* animals treated with or without *fasn-1* RNAi and 4.5 mM phenformin from L4 of development to AD3. Lysate from approximately 1,000 animals was harvested for each condition. Due to the developmental delay in *crn-3(ok2269)* mutants, redox measurements were performed a day after wild-type for stage-matching. Data are compiled from 3 independent biological replicates. c) Quantification of GSSG/GSH ratios using a fluorescent redox-sensitive *rpl-17p::Grx1-roGFP2* reporter expressed in wild-type and *crn-3(ok2269)* animals treated with or without *fasn-1* RNAi and 4.5 mM phenformin from L4 of hatching to AD5. Experiment was performed in 2 biological replicates with a total of 60 animals imaged per condition. All error bars represent mean ± SEM unless otherwise noted. Significance was assessed in (a to c) using a 2-way ANOVA with interaction analysis and Sidak correction of multiple comparison testing (see Supplementary Data Tables). Corrected *P*-values are shown in figure. ^ns^*P* > 0.05, *P < 0.05, **P < 0.01, ***P < 0.001, ****P < 0.0001.

Phenformin, *fasn-1* RNAi, and combined treatment caused NADPH/NADP+ ratios to increase in both wild-type and *crn-3* mutant animals (Fig. 7a), consistent with the accumulation of NADPH described in Ahsan et al. (2026). When investigating NADH/NAD+ ratios in wild-type animals, we observed that phenformin caused a nonsignificant increase while *fasn-1* RNAi resulted in a nonsignificant decrease in the NADH/NAD+ ratio from baseline (Fig. 7b). The combination of both treatments resulted in a significant increase in the NADH/NAD+ ratio compared to *fasn-1* RNAi alone, restoring the NADH/NAD+ ratio to baseline (Fig. 7b). Thus, phenformin synergizes with *fasn-1* RNAi, causing the accumulation of NADH in wild-type animals.

Meanwhile, results measuring the NADH/NAD+ ratio in *crn-3* mutants revealed a very different pattern. In *crn-3* mutants, phenformin treatment resulted in a significant decrease in the NADH/NAD+ ratio, as did *fasn-1* RNAi alone (Fig. 7b). Notably, the combined treatments did not result in a synergistic elevation of the NADH/NAD+ ratio in *crn-3* mutants (Fig. 7b). Together, these results suggest that the *crn-3* mutation may improve survival outcomes by increasing the animal's tolerance of high levels of NADPH and NADH caused by combined phenformin and *fasn-1* RNAi rather than preventing their accumulation. It is also notable that the effects of phenformin and *fasn-1* RNAi on NADH/NAD+ ratio dynamics vary significantly between strains, suggesting that *crn-3* deficiency can fundamentally alter the cellular response to exogenous reductive stressors.

To determine whether inhibition of *crn-3* may prevent GSH reductive stress, we genetically crossed *crn-3(ok2269)* animals with animals expressing an in vivo GSH/GSSH reporter. We observed a synthetic decrease in GSSG/GSH ratios upon combined phenformin and *fasn-1* RNAi in the control strain, consistent with GSH reductive stress (Fig. 7c). Notably, GSSG/GSH ratios were significantly increased in *crn-3* mutant worms across all treatment conditions when compared to control animals (Fig. 7c). However, a similar decreasing trend of GSSG/GSH ratios was still observed in *crn-3* animals treated with combined phenformin and *fasn-1* RNAi (Fig. 7c). Together is suggests that the *crn-3* mutation shifts the glutathione redox pools toward a more oxidized state, potentially alleviating toxic GSH accumulation.

### Inhibiting rRNA synthesis activates nuclear RNAi pathway

Other groups have shown that erroneous processing of rRNA transcripts caused by *crn-3*, *bud-23*, *riok-1*, or *t22h9.1* knockdown can activate a nuclear RNAi-mediated pathway in *C. elegans* that inhibits RNA polymerase I activity via the generation of ribosomal silencing rRNAs (risiRNAs) (Zhu et al. 2018; Liao et al. 2021). Thus, we wondered whether this negative feedback loop is a signaling pathway through which organisms may inhibit rRNA synthesis to protect themselves against reductive stress.

The generation of risRNAs involves RNA-dependent RNA polymerases (RdRPs) including *rrf-2* and *rrf-1* which are activated when erroneous rRNA transcripts accumulate in the nucleolus (Zhu et al. 2018; Liao et al. 2021). Nuclear argonauts like *nrde-3* aid in the translocation of risiRNAs into the nucleus where they inhibit RNA polymerase I activity (Zhu et al. 2018; Liao et al. 2021). To determine how nuclear RNAi signaling may be affected by phenformin and *fasn-1* RNAi, we analyzed RNA sequencing data and observed a significant increase in *rrf-2* and *nrde-3* mRNA expression in phenformin-treated animals (Fig. 8, a and b). Intriguingly *fasn-1* RNAi completely blocked phenformin-induced overexpression of these genes (Fig. 7, a and b).

**Fig. 8.**
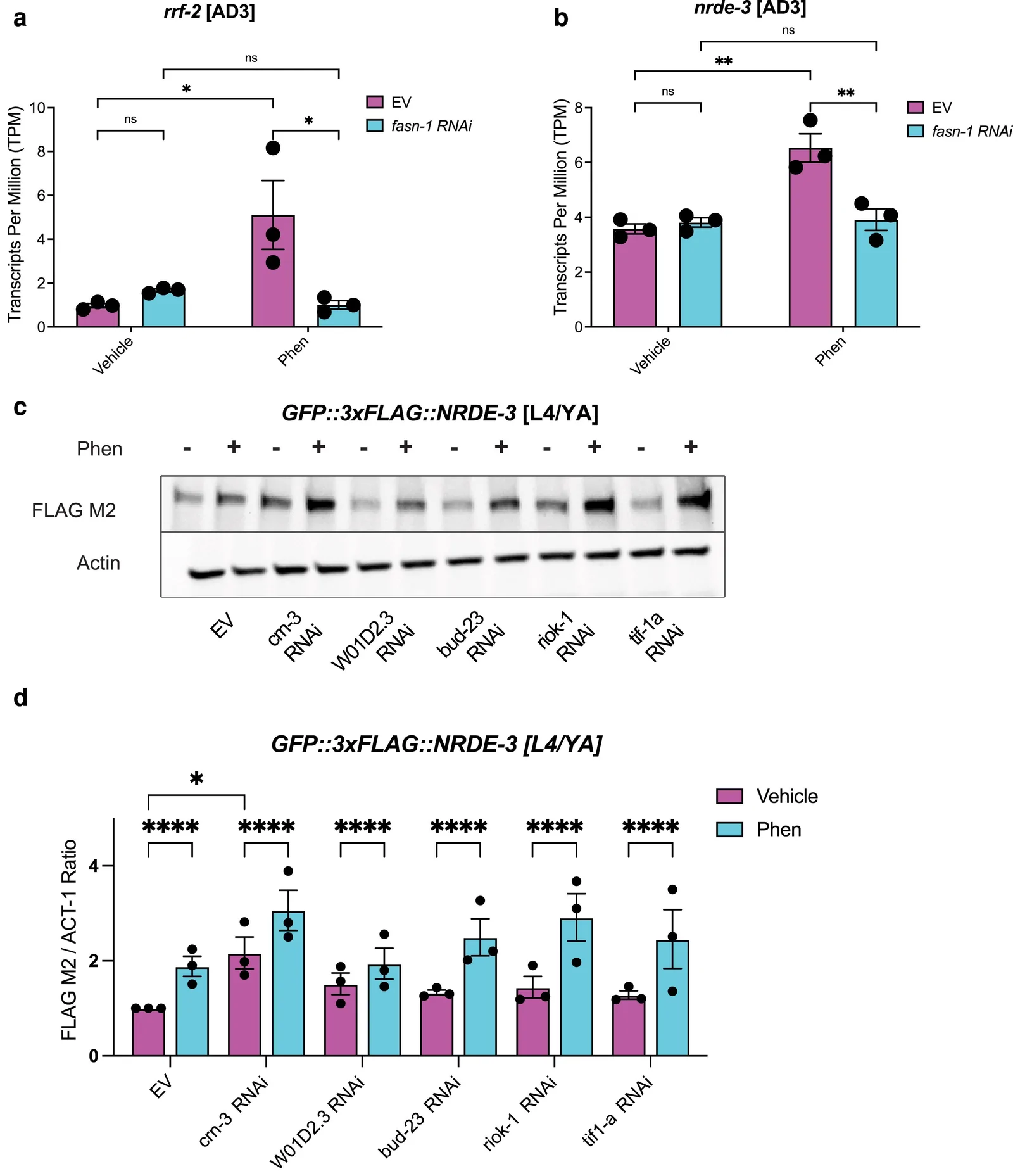
Phenformin and *crn-*3 RNAi activate *fasn-1-*dependent nuclear RNAi signaling pathway. a and b) Abundance of (a) *rrf-2* and (b) *nrde-*3 mRNA transcripts in N2 wild-type worms fed EV or *fasn-1* RNAi and treated with vehicle or 4.5 mM phenformin from L4 of hatching to AD3. Results are compiled from 3 independent biological replicates. c) Representative immunoblot showing *NRDE-3* protein expression in *GFP:: 3xFLAG::NRDE-3* animals fed RNAi targeting rRNA processing genes and treated with or without 4.5 mM phenformin from L1 of hatching to L4/YA. d) Quantification of *NRDE-*3 immunoblotting. Results are inclusive of 3 independent biological replicates. All error bars represent mean ± SEM unless otherwise noted. Significance was assessed in (a and b) using a 2-way ANOVA with interaction analysis and Sidak correction of multiple comparison testing (see Supplementary Data Tables). d) Significance was assessed using a 2-way ANOVA without interaction analysis and Sidak correction of multiple comparison testing (see Supplementary Data Tables). Corrected *P*-values are shown in figure. ^ns^*P* > 0.05, *P < 0.05, **P < 0.01, ***P < 0.001, ****P < 0.0001.

We confirmed that phenformin caused an increase in *NRDE-3* protein expression via immunoblotting (Fig. 8c; see Supplementary Immunoblots). Notably, *crn-3* RNAi caused *NRDE-3* expression to dramatically increase in both the vehicle- and phenformin-treated conditions when compared to the EV controls (Fig. 8c; see Supplementary Immunoblots). RNAi knockdown of *bud-23*, *riok-1*, and *tif-1a* resulted in a slight, yet insignificant, increase in *NRDE-3* expression in both the vehicle- and phenformin-treated conditions compared to controls (Fig. 8c; see Supplementary Immunoblots). These results suggest that phenformin activates a nuclear RNAi pathway that is *fasn-1-*dependent and *crn-3* inhibition may have an additive effect when combined with phenformin. It remains unclear what the exact downstream targets of this nuclear RNAi pathway are and whether activation of this pathway is required for *crn-3-*mediated resistance to phenformin-induced reductive stress.

## Discussion

In this study, we continued our previous investigation of biguanide-induced reductive death, which we found occurs upon the inhibition of de novo fatty acid synthesis. This reductive death phenotype is due to a decrease in NADH, NADPH, and GSH redox buffering capacity, leading to a toxic accumulation of these reducing factors (Ahsan et al. 2026). Seeking potential ways to mitigate reductive stress, we discovered that deficiency in several rRNA transcription and maturation factors averts biguanide-induced reductive death in *fasn-1-*deficient *C. elegans*. Meanwhile, genetic manipulations that enhanced rRNA synthesis synergized with phenformin and *fasn-1* deficiency to aggravate hypodermal stress markers associated with phenformin-induced reductive death. Notably, *crn-3* deficiency significantly altered redox dynamics, rewiring the worm's response to the accumulation of reducing equivalents.

Our findings support existing research identifying differential rRNA modification as a key regulator of stress resistance and longevity. For example, preventing the methylation of 18S rRNA by *dimt-1* at adenosines 1,735 and 1,736 in *C. elegans* is sufficient to not only extend lifespan up to 33%, but can also improve resistance to UV irradiation, and heat stress via selective translation of genes involved in longevity, degradation pathways, detoxification, glutathione metabolism, and energy production (Rothi et al. 2025). Similarly, mutation of *metl-5* prevents the methylation of adenosine 1,717 on 18S rRNA, resulting in increased resistance to oxidative, cold, severe heat shock, and UV irradiation thanks to the reduced translation of the gene *cyp-29A3* (Liberman et al. 2020). These findings highlight the importance of ribosome diversity in regulating cellular responses to stressors and other stimuli. Variation in rRNA processing is a major source of ribosome diversity and plasticity that is often underappreciated. Thus, ribosome heterogeneity provides an added level of posttranscriptional gene regulation by enabling selective loading and translation of mRNA transcripts (Trahan and Oeffinger 2023).

Quality control of RNA in the nucleus and cytoplasm involves the RNA exosome complex consisting of 9 subunits (exosc-1-9) that make up the exosome's inert core, and 2 catalytic subunits (*exosc-10* and *DIS3*) play a dual role in the degradation of erroneous mRNA and rRNA transcripts as well as the processing of rRNA (Puno et al. 2019). For example, knockdown of EXOSC2 and EXOSC5 through EXOSC10 results in impaired processing of 5.8S and 18S rRNA (Tafforeau et al. 2013; Feng et al. 2025). Thus, targeting specific subunits of the RNA exosome may also be a source of ribosome diversity and a way to boost an organism's resistance to stress.

Previous work has shown that inhibiting nucleolar and ribosomal activity improves stress resistance by changing the lipidome (Sharifi et al. 2024). Inducing nucleolar stress activates transcription factor *pha-4* and promotes lipid accumulation to improve starvation resistance in *C. elegans* (Wu et al. 2018; Feng et al. 2025). Similarly, inhibition of tRNA genes also promotes lipid accumulation and starvation resistance by activating AMPK signaling pathways (Webster et al. 2017). The type of fatty acids that are produced may also dictate resistance to specific stressors. For example, impairing *crn-3* may enhance the production of oleic acid, which improves oxidative stress resistance in *C. elegans* (Feng et al. 2025).

We observed that *crn-3-*deficient animals overexpress FASN-1 protein. In the context of reductive stress, enhanced *fasn-1* activity is likely to be advantageous as de novo fatty acid synthesis is an essential pathway through which NADPH is consumed. Thus, *crn-3* deficiency and reduced rRNA synthesis may beneficially increase redox buffering capacity through enhanced *fasn-1* activity. However, in this study, we leverage both RNAi and AID knockdown to show that inducing nucleolar stress promotes stress resistance even in the absence of *fasn-1*. Thus, it's possible that inhibiting rRNA processing genes may also protect against reductive stress in a manner that is independent of their roles in lipid metabolism.

The reducing equivalent NADH is closely tied to cellular energetics through complex I of the electron transport chain (ETC). Complex I is a NADH:uniquinone oxidoreductase that utilizes NADH as an electron donor, effectively powering the process of oxidative phosphorylation (Yang et al., 2025). In times of high energetic demand, increased flux through the ETC may decrease mitochondrial NADH pools. Conversely, low energetic demand and reduced mitochondrial activity or mitochondrial dysfunction may cause NADH levels to increase (Yang et al. 2025).

It is estimated that 80% of all RNA species produced in the cell are rRNAs. Inhibiting rRNA synthesis could reduce energetic demand, thus causing NADH levels to rise. As such, we might expect that a decrease in ETC activity could increase reducing equivalents like NADH. Conversely, an increase in energy consumption could shift the redox balance toward a more oxidized state. Further work could be done to better understand how energetically expensive growth-promoting processes may directly impact redox status.

We observed an elevation of basal NADPH/NADP+ and NADH/NAD+ ratios in *crn-3* mutants compared to wild-type animals. This could be consistent with the role of *crn-*3 in ribosomal biogenesis. A decrease in rRNA production might correspond with a decrease in ETC activity and reduced consumption of NADH. Notably, this accumulation of NADH levels did not lead to reductive death in *crn-3* mutants. We also observed that inhibiting the energetically expensive process of translation by targeting elongation and initiation did not rescue reductive death. This points toward something unique about inhibiting rRNA synthesis that protects against reductive stress independent of downstream inhibition of translation or energetic demand. Together, these findings suggest that inhibiting *crn-3* and potentially rRNA synthesis improves survival outcomes not by preventing the accumulation of reducing equivalents, but rather by activating downstream signaling pathways that promote tolerance of NADH reductive stress. Interestingly, we find that administration of phenformin and inhibition of rRNA processing genes may activate nuclear RNAi-related genes *nrde-3* and *rrf-2*. Whether nuclear RNAi is necessary for reductive stress resistance is yet to be determined and warrants further investigation.

In our investigation, we observed that phenformin administration caused distinct changes in nucleolar morphology. Phenformin alone significantly reduced the ratio of nucleolar to nuclear size in hypodermal cells. However, this was reversed in the absence of *fasn-1,* causing a potential aberrant increase in downstream translation efficiency. The discovery that inhibition of *fasn-1* protects rRNA biogenesis despite phenformin treatment was intriguing as biguanides may promote longevity by inhibiting global levels of translation as evidenced by a decrease in the polysome to monosome ratio (Ahsan et al. 2026). These findings point toward rRNA synthesis as a possible mechanism through which biguanides could be modulating translation.

To our knowledge, this is the first paper to identify biguanides and *fasn-1* as potential regulators of nucleolar size and activity in *C. elegans*. In human MCF-7 breast cancer cells, metformin reduces rRNA transcription by activating AMPK and inhibiting succinate production via the TCA cycle to epigenetically regulate rDNA expression (Tanaka et al. 2019). Biguanide-mediated activation of AMPK has also been shown to increase the expression of nucleolin and prevent the nuclear localization of fibrillarin, proteins that are known to be negative and positive regulators of rRNA synthesis, respectively (Kodiha et al. 2014). It has also been suggested that AMPK can directly phosphorylate *tif-1a* to suppress its activity (Hoppe et al. 2009). Interestingly, our previous work shows that loss of *fasn-1* activity prevents phenformin-mediated phosphorylation of *aak-2*—the *C. elegans* homolog of human AMPK (Ahsan et al. 2026). This seemingly correlates with the inability to reduce nucleolar size in *fasn-1-*deficient animals treated with phenformin. Thus, future work could investigate whether phenformin-mediated regulation of nucleolar size relies upon a conserved AMPK-dependent pathway in *C. elegans*.

Other studies have linked cellular energetics to rRNA synthesis via the energy-dependent Nucleolar Silencing Complex (eNOSC), which is partially comprised of the NAD+-dependent histone deacetylase SIRT1. Thus, in cases of low NAD+ levels, such as glucose deprivation, eNOSC epigenetically silences rDNA transcription (Murayama et al. 2008). This work establishes a potential link between NADH reductive stress and enhanced ribosomal biogenesis where low NAD+ availability could prevent eNOSC-mediated epigenetic silencing of rDNA.

The nutrient sensor mTOR has also been shown to regulate RNA and rRNA metabolism. Mutating *rsks-1*or *rict-1* can prevent the nucleolar localization of some RNA exosome subunits and vice versa where inhibition of RNA exosome subunits downregulates translation via reduced TOR signaling (Feng et al. 2025). Others have asserted that boosting mTOR activity promotes *tif-1a* association with rDNA, increasing rRNA synthesis (Mayer et al. 2004). Thus, multiple energy sensing pathways may play a key role in regulating rRNA synthesis during phenformin-induced reductive stress.

The mechanisms through which inhibition of *fasn-1* may increase nucleolar activity and translation in *C. elegans* is also unknown. Studies in cellular models have identified the mammalian homolog *FASN* as a regulator of rRNA biogenesis and nucleolar size. It has been proposed that *fasn-1* inhibition causes the accumulation of the intermediate metabolite malonyl-CoA, promoting the malonylation of lysine residues on histones and nucleolin (Zhang et al. 2023; Sun et al. 2024). These posttransitional modifications can drive ribosomal biogenesis as evidenced by assessments of nucleolar size, qRT-PCR, and proliferation and growth of hepatocellular carcinoma (Zhang et al. 2023; Sun et al. 2024). These findings link *fasn-1* to enhanced rRNA synthesis through malonyl-CoA in cancer models, a key metabolite involved in the regulation of fatty acid metabolism as well as nutrient sensing.

Investigation into how biguanides regulate rRNA synthesis could be important to our understanding of the safety and efficacy of these drugs as potential anti-aging interventions. Transcription of rRNA is negatively regulated by nucleolin (*ncl-1*), and thus *ncl-1* null mutants exhibit increased nucleolar size, higher levels of rRNA, and translate more protein (Frank and Roth 1998). Unleashing rRNA synthesis by knocking down *ncl-1* blocks several lifespan-extending paradigms associated with reduced TORC activity, insulin signaling, reproduction, and mitochondrial activity in *C. elegans* (Tiku et al. 2017). Metformin-lifespan extension has been shown to act through a number of these distinct geroprotective pathways (Soukas et al., 2019). Therefore, it's unsurprising that reduced rRNA synthesis may be necessary for achieving maximum biguanide-mediated lifespan extension.

We demonstrate that increasing rRNA synthesis results in heightened sensitivity to the effects of combined phenformin and *fasn-1* deficiency. Notably, the stress reporter for *nlp-*29 was activated in *ncl-1-*deficient animals upon treatment with phenformin with or without genetic inhibition of *fasn-1*. Combining all 3 conditions resulted in synergistic overexpression of *nlp-29* supporting our hypothesis that enhanced rRNA synthesis hypersensitizes animals to the potentially detrimental effects of phenformin-induced reductive stress. Together, these findings suggest that reductions in nucleolar activity may be necessary for the detoxification of the drug, highlight the regulatory role of the nucleolus in aging, and point to nucleolar activity being a biological switch that determines whether phenformin is a life-extending or life-shortening intervention.

## Limitations

Future work should also be done to determine if alterations to rRNA biogenesis can rescue toxicity associated with other paradigms of reductive stress not involving phenformin or *fasn-1* deficiency. Additionally, this study focuses on inhibiting ribosomal biogenesis by targeting of RNA polymerase I activity and RNA polymerase I-transcribed rRNAs. It would be interesting to determine whether interrupting ribosome biogenesis via the perturbation of RNA polymerase III-mediated 5S rRNA and tRNA synthesis or select ribosomal proteins may similarly protect against reductive stress. And while nucleolar size is a proxy for rRNA synthesis, measuring the cleavage, methylation, and modification of rRNA transcripts should also be studied to understand how biguanides are altering ribosomal biogenesis.

## Conclusions

Overall, our findings suggest that restricting pre-rRNA transcription or altering its modification and processing may serve to detoxify reductive stress. Targeting this highly conserved pathway may beneficially increase buffering capacity during insults to redox homeostasis. As such, modulating rRNA biogenesis could hold therapeutic potential for addressing diseases such as cancers, cardiovascular disease, and other metabolic ailments associated with reductive stress.

## Supplementary Material

iyag214_Supplementary_Data

## Data Availability

Any strains or plasmids used in this study are available upon request. All data necessary for confirming the conclusion of the article can be located within the article, figures, and tables. All RNA-sequencing data have been uploaded to the NCBI Gene Expression Omnibus (GEO) under accession number GSE277946. Additionally, images of uncropped western blot membranes have been made available in the Supplementary data files. Supplemental material available at GENETICS online.
