# Supplementary material for: Inhibiting ribosomal RNA synthesis in C. elegans protects against reductive stress during de novo fatty acid synthesis deficiency
Source: Genetics. 2026 Aug 11;234(3):iyag214. doi: 10.1093/genetics/iyag214 (PMC13732720; doi:10.1093/genetics/iyag214)
Supplement: Supplementary file 1 [file iyag214_supplementary_data.zip › Figure_S1_GENETICS-2026-309627.pdf]

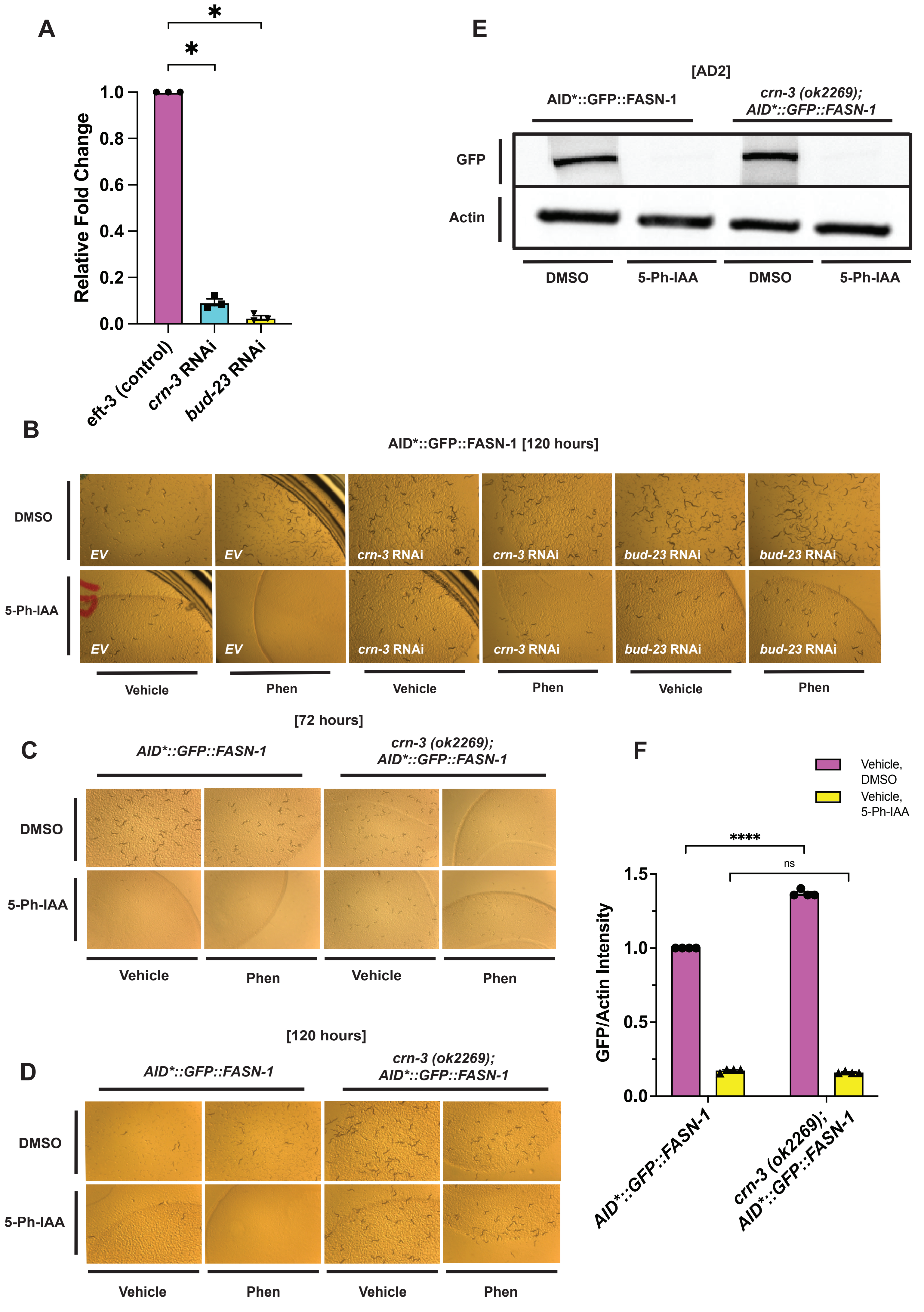
A
*
*
1.0
0.8
0.6
0.4
0.2
0.0
Relative Fold Change
eft-3 (control)
crn-3 RNAi
bud-23 RNAi
E
[AD2]
AID*::GFP::FASN-1
crn-3 (ok2269);
AID*::GFP::FASN-1
GFP
Actin
DMSO
5-Ph-IAA
DMSO
5-Ph-IAA
B
AID*::GFP::FASN-1 [120 hours]
DMSO
5-Ph-IAA
EV
EV
crn-3 RNAi
crn-3 RNAi
bud-23 RNAi
bud-23 RNAi
Vehicle
Phen
Vehicle
Phen
Vehicle
Phen
[72 hours]
C
AID*::GFP::FASN-1
crn-3 (ok2269);
AID*::GFP::FASN-1
DMSO
5-Ph-IAA
Vehicle
Phen
Vehicle
Phen
F
Vehicle, DMSO
Vehicle, 5-Ph-IAA
****
ns
1.5
1.0
0.5
0.0
GFP/Actin Intensity
AID*::GFP::FASN-1
crn-3 (ok2269);
AID*::GFP::FASN-1
[120 hours]
D
AID*::GFP::FASN-1
crn-3 (ok2269);
AID*::GFP::FASN-1
DMSO
5-Ph-IAA
Vehicle
Phen
Vehicle
Phen
