# Supplementary material for: Inhibiting ribosomal RNA synthesis in C. elegans protects against reductive stress during de novo fatty acid synthesis deficiency
Source: Genetics. 2026 Aug 11;234(3):iyag214. doi: 10.1093/genetics/iyag214 (PMC13732720; doi:10.1093/genetics/iyag214)
Supplement: Supplementary file 1 [file iyag214_supplementary_data.zip › Figure_S2_GENETICS-2026-309627.pdf]

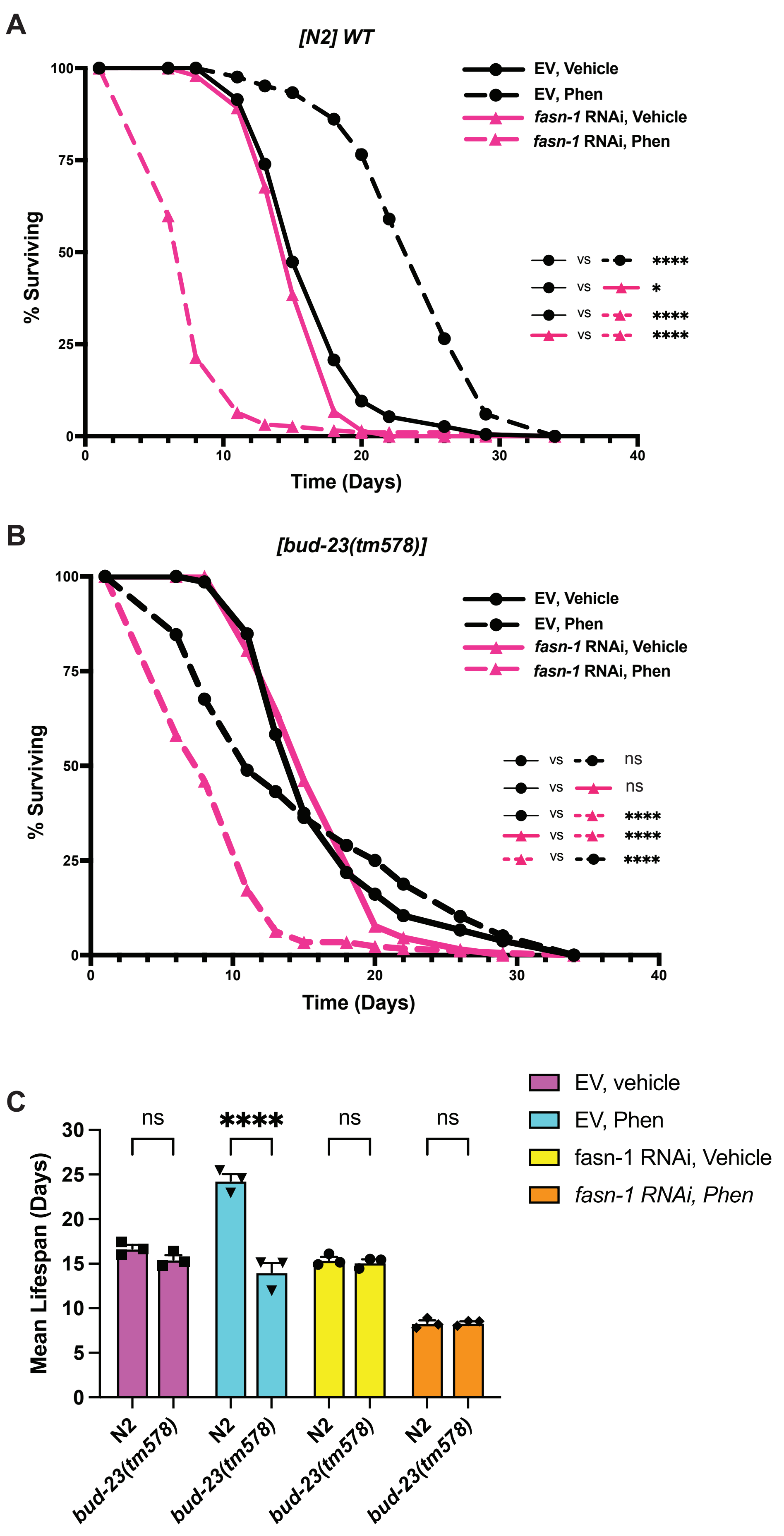
A
[N2] WT
% Surviving
Time (Days)
EV, Vehicle
EV, Phen
fasn-1 RNAi, Vehicle
fasn-1 RNAi, Phen
vs ****
vs *
vs ****
vs ****
B
[bud-23(tm578)]
% Surviving
Time (Days)
EV, Vehicle
EV, Phen
fasn-1 RNAi, Vehicle
fasn-1 RNAi, Phen
vs ns
vs ns
vs ****
vs ****
vs ****
C
Mean Lifespan (Days)
ns
****
ns
ns
N2
bud-23(tm578)
EV, vehicle
EV, Phen
fasn-1 RNAi, Vehicle
fasn-1 RNAi, Phen
