# Supplementary material for: Inhibiting ribosomal RNA synthesis in C. elegans protects against reductive stress during de novo fatty acid synthesis deficiency
Source: Genetics. 2026 Aug 11;234(3):iyag214. doi: 10.1093/genetics/iyag214 (PMC13732720; doi:10.1093/genetics/iyag214)
Supplement: Supplementary file 1 [file iyag214_supplementary_data.zip › Figure_S3_GENETICS-2026-309627.pdf]

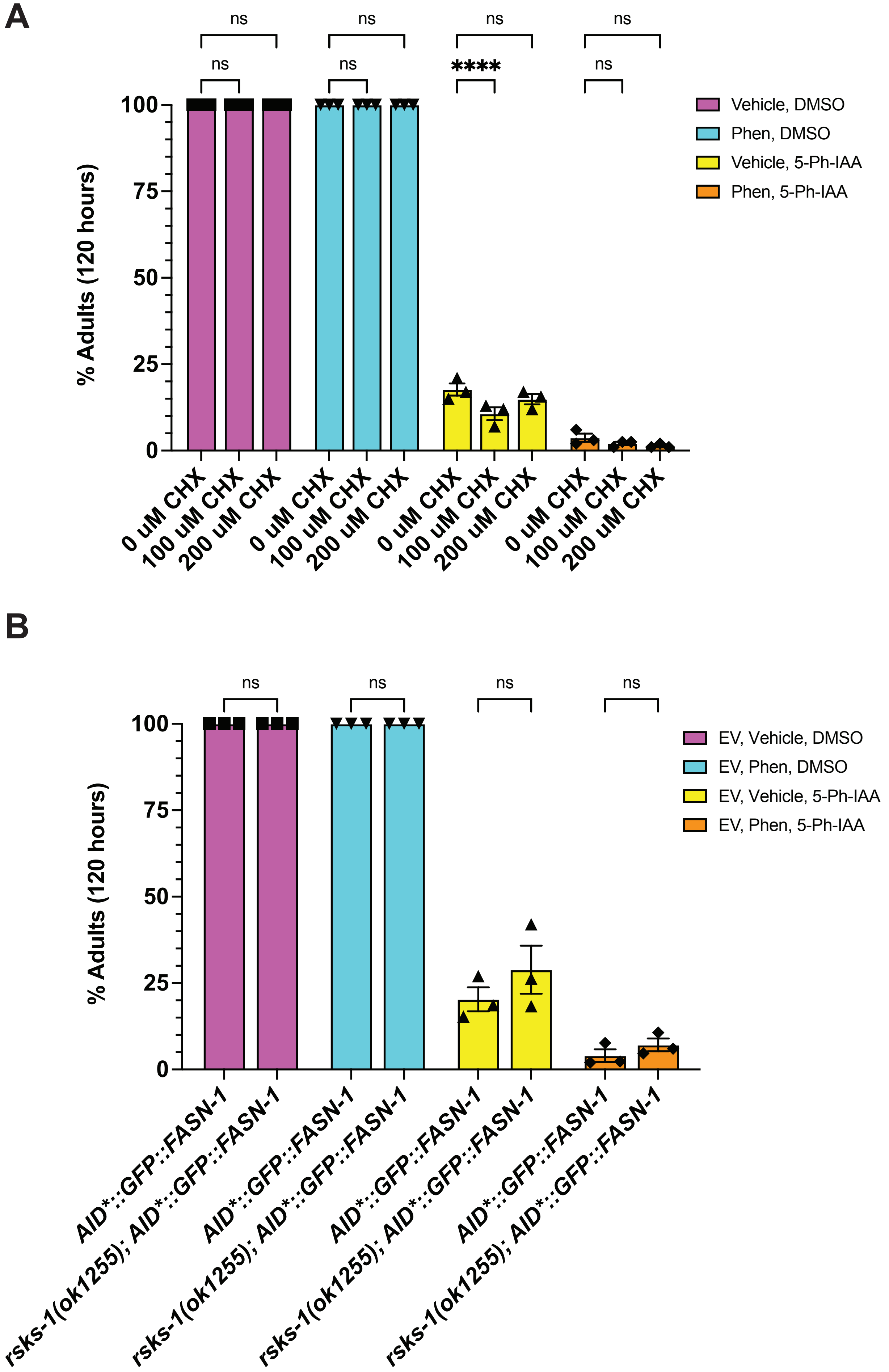
A
ns
ns
ns
ns
ns
ns
****
ns
100
75
50
25
0
% Adults (120 hours)
Vehicle, DMSO
Phen, DMSO
Vehicle, 5-Ph-IAA
Phen, 5-Ph-IAA
0 uM CHX
100 uM CHX
200 uM CHX
0 uM CHX
100 uM CHX
200 uM CHX
0 uM CHX
100 uM CHX
200 uM CHX
0 uM CHX
100 uM CHX
200 uM CHX
B
ns
ns
ns
ns
100
75
50
25
0
% Adults (120 hours)
EV, Vehicle, DMSO
EV, Phen, DMSO
EV, Vehicle, 5-Ph-IAA
EV, Phen, 5-Ph-IAA
AID*::GFP::FASN-1
rsks-1(ok1255); AID*::GFP::FASN-1
AID*::GFP::FASN-1
rsks-1(ok1255); AID*::GFP::FASN-1
AID*::GFP::FASN-1
rsks-1(ok1255); AID*::GFP::FASN-1
AID*::GFP::FASN-1
rsks-1(ok1255); AID*::GFP::FASN-1
