# Supplementary material for: Inhibiting ribosomal RNA synthesis in C. elegans protects against reductive stress during de novo fatty acid synthesis deficiency
Source: Genetics. 2026 Aug 11;234(3):iyag214. doi: 10.1093/genetics/iyag214 (PMC13732720; doi:10.1093/genetics/iyag214)
Supplement: Supplementary file 1 [file iyag214_supplementary_data.zip › Figure_S4_GENETICS-2026-309627.pdf]

A

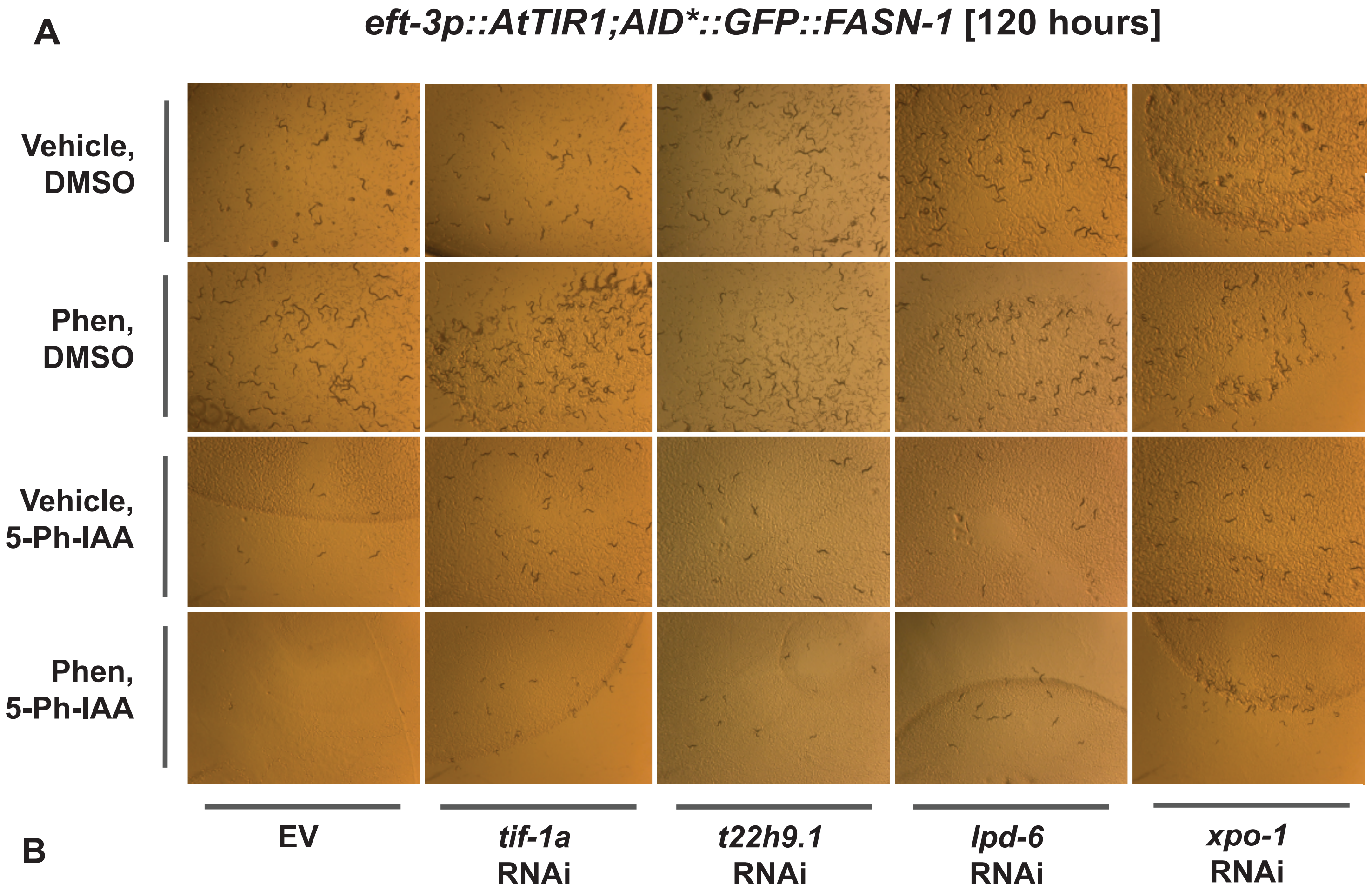

eft-3p::AtTIR1;AID*::GFP::FASN-1 [120 hours]
Vehicle, DMSO
Phen, DMSO
Vehicle, 5-Ph-IAA
Phen, 5-Ph-IAA
EV
tif-1a RNAi
t22h9.1 RNAi
lpd-6 RNAi
xpo-1 RNAi

B

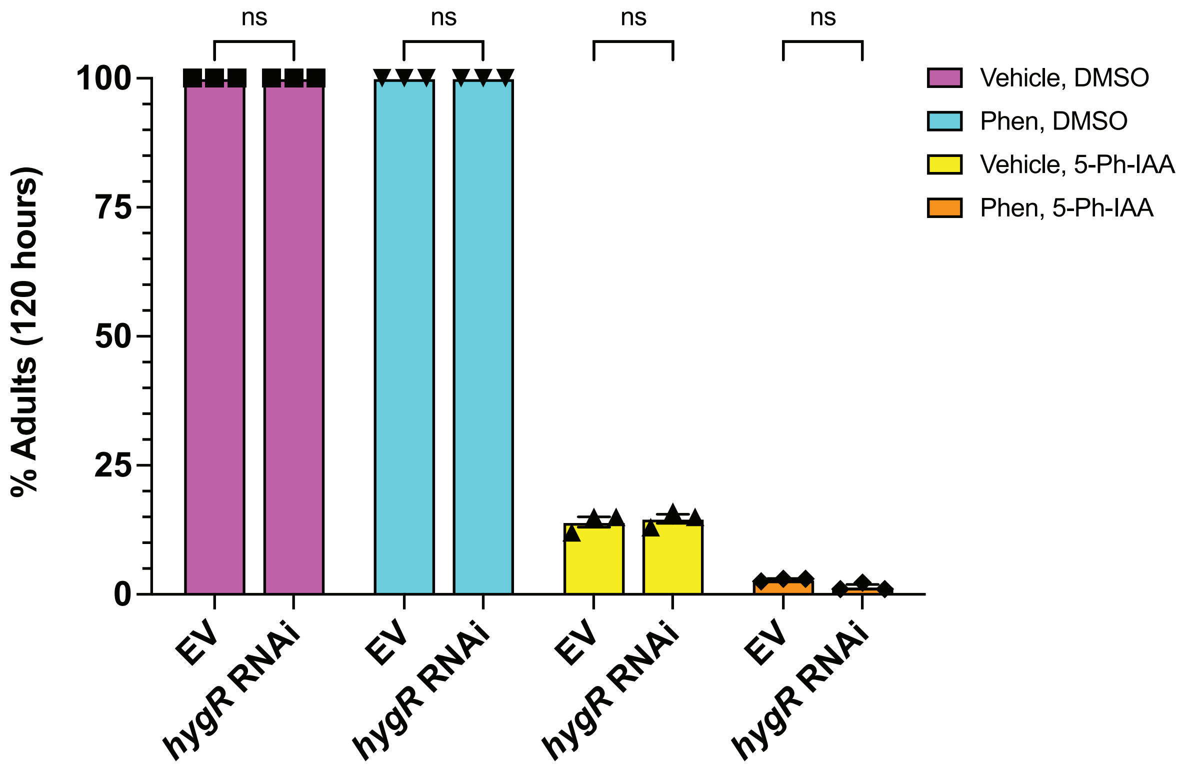

ns
ns
ns
ns
% Adults (120 hours)
100
75
50
25
0
EV
hygR RNAi
EV
hygR RNAi
EV
hygR RNAi
EV
hygR RNAi
Vehicle, DMSO
Phen, DMSO
Vehicle, 5-Ph-IAA
Phen, 5-Ph-IAA
