# Supplementary material for: Inhibiting ribosomal RNA synthesis in C. elegans protects against reductive stress during de novo fatty acid synthesis deficiency
Source: Genetics. 2026 Aug 11;234(3):iyag214. doi: 10.1093/genetics/iyag214 (PMC13732720; doi:10.1093/genetics/iyag214)
Supplement: Supplementary file 1 [file iyag214_supplementary_data.zip › Supplemental_Figure_Legends_GENETICS-2026-309627.docx]

**Figure S1. Knockdown of *crn-3* and *bud-3* via Auxin Degradable Degron System or RNA Interference Protects Against Phenformin-Induced Reductive Stress in *fasn-1* Depleted Animals**

**(A)** Quantification of RT-qPCR measuring RNAi knockdown efficiency of *crn-3* and *bud-23* mRNA transcripts in wild-type animals. *eft-3* was used as a housekeeping gene for normalization. Results are compiled from 3 independent biological replicates. **(B)** Representative images of developmental assay performed on *AID*::GFP::FASN-1* animals treated or without 4.5 mM phenformin and 5 µM 5-Ph-IAA and fed EV, *crn-3*, or *bud-23* RNAi. **(C - D**) Representative images of developmental assay performed on control *AID*::GFP::FASN-1* or *crn-3(2269); AID*::GFP::FASN-1* animals after (C) 72 hours of treatment or (D)120 hours of treatment from L1 of hatching. **(E)** Immunoblotting for FASN-1 protein in *eft-3p::AtTIR-1(F79G)::mRuby;3xFLAG::AID*::GFP::FASN-1 and crn-3(ok2269)*; *eft-3p::AtTIR-1(F79G)::mRuby;3xFLAG::AID*::GFP::FASN-1* animals treated with or without 4.5 mM phenformin and 5 µM 5-Ph-IAA. Results are representative of 4 independent biological replicates. Note that control *AID*::GFP::FASN-1* were treated with drugs post-developmentally from L4 of hatching to AD2 due to 5-Ph-IAA treatment being toxic when administered from L1 of hatching. Meanwhile *crn-3(ok2269)::AID*::GFP::FASN-1* animals were treated from L1 of hatching to AD2. **(F)** Quantification of FASN-1 protein expression in control versus *crn-*3 mutants via immunoblotting. Results are compiled from 4 independent biological replicates. All error bars represent mean +/- SEM unless otherwise noted. Significance was assessed in **(A)** using a one-way with Sidak correction of multiple comparison testing and **(F)** using a two-way ANOVA with interaction analaysis and Sidak correction of multiple comparison testing (see Supplemental Data Tables). Corrected p-values are shown in figure. ns p > 0.05, * p < 0.05, ** p < 0.01, *** p < 0.001, **** p < 0.0001.

**Alt Text:** Graphs and images labeled A-F. A is a bar graph of mRNA expression of *crn-3* and *bud-*23, showing effective RNAi knockdown with significance markers. B, C, and D show images of agar plates containing *C. elegans* showing their developmental stage at different timepoints and across different RNAi conditions and genotypes. E and F are an immunoblot and bar graph visualizing auxin induced degradation of *FASN-1* protein expression in wild-type versus *crn-3* mutant animals.

**Figure S2. Genetic Mutation of *bud-23* Does not Reverse Post-Developmental Reductive Death**

**(A-B)** Lifespan analyses of (A) wild-type and (B) *bud-23(tm578)* animals treated with or without 4.5 mM phenformin and *fasn-1* RNAi from L4 of hatching. **(C)** Mean lifespans of wild-type animals vs *bud-23(tm578)* animals. Data represents 3 independent biological replicates performed in technical triplicate. All error bars represent mean +/- SEM unless otherwise noted. All mutant animals were backcrossed 4-6 times to wild-type N2 animals to remove potential background mutations. Significance was assessed in **(A-C)** using log rank analyses and cox proportional hazard regression (see Supplementary Data Tables). Corrected p-values are shown in figure. ns p > 0.05, * p < 0.05, ** p < 0.01, *** p < 0.001, **** p < 0.0001.

**Alt Text:** Graphs depicting lifespan data labeled A-C with significance markers. A and B depict lifespan curves for wild-type and *bud-23 (tm578)* mutants. C is a bar graph comparing the mean lifespan of wild-type and *bud-23 (tm578)* mutants.

**Figure S3: Inhibiting Translation Does Not Rescue Phenformin-Mediated Reductive Stress in *fasn-1* Deficient Animals**

(A) Quantification of developmental assay in *eft-3p::AtTIR-1(F79G)::mRuby;3xFLAG::AID*::GFP::FASN-1* animals treated with or without 4.5 mM phenformin, 5 µM 5-Ph-IAA, and cycloheximide. Results are compiled from 3 independent biological replicates. (**B)** Quantification of developmental assay in *eft-3p::AtTIR-1(F79G)::mRuby;3xFLAG::AID*::GFP::FASN-1* and *rsks-1(ok1255);eft-3p::AtTIR-1(F79G)::mRuby;3xFLAG::AID*::GFP::FASN-1* animals treated with or without 4.5 mM phenformin and 5 µM 5-Ph-IAA. All error bars represent mean +/- SEM unless otherwise noted. Significance was assessed in **(A, E)** using a two-way ANOVA with interaction analysis and Sidak correction of multiple comparison testing (see Supplemental Data Tables). Corrected p-values shown in figure. ns p > 0.05, * p < 0.05, ** p < 0.01, *** p < 0.001, **** p < 0.0001.

**Alt Text:** Graphs labeled A-B with statistical markers measuring the percentage of the population surviving to adulthood, showing that outcomes are not improved by inhibiting translation.

**Figure S4. RNAi Knockdown of rRNA Biogenesis Genes Protects Against Phenformin-Induced Reductive Stress**

**(A)** Representative images of developmental assay performed on *AID*::GFP::FASN-1* animals fed RNAi expressing bacteria and treated with or without 5 µM 5-Ph-IAA and 4.5 mM phenformin. Images were taken 120 hours after treatment from L1 of hatching. **(B)** . Quantification of larval development assay in *eft-3p::AtTIR-1(F79G)::mRuby;3xFLAG::AID*::GFP::FASN-1* animals treated with or without 4.5 mM phenformin, 5 µM 5-Ph-IAA, and a non-targeting hygR RNAi. All error bars represent mean +/- SEM unless otherwise noted. Significance was assessed in **(B)** using a two-way ANOVA with interaction analysis and Sidak correction of multiple comparison testing (see Supplemental Data Tables). Corrected p-value shown in figure.. ns p > 0.05, * p < 0.05, ** p < 0.01, *** p < 0.001, **** p < 0.0001.

**Alt Text:** Images and graphs labeled A-B. A shows agar plates containing *C. elegans* fed various rRNA-targeting RNAi. B is a bar graph measuring the development of *C, elegans*.
