# Supplementary material for: Inhibiting ribosomal RNA synthesis in C. elegans protects against reductive stress during de novo fatty acid synthesis deficiency
Source: Genetics. 2026 Aug 11;234(3):iyag214. doi: 10.1093/genetics/iyag214 (PMC13732720; doi:10.1093/genetics/iyag214)
Supplement: Supplementary file 1 [file iyag214_supplementary_data.zip › Supplemental_Immunoblots_GENETICS-2026-309627.pdf]

Membrane 1

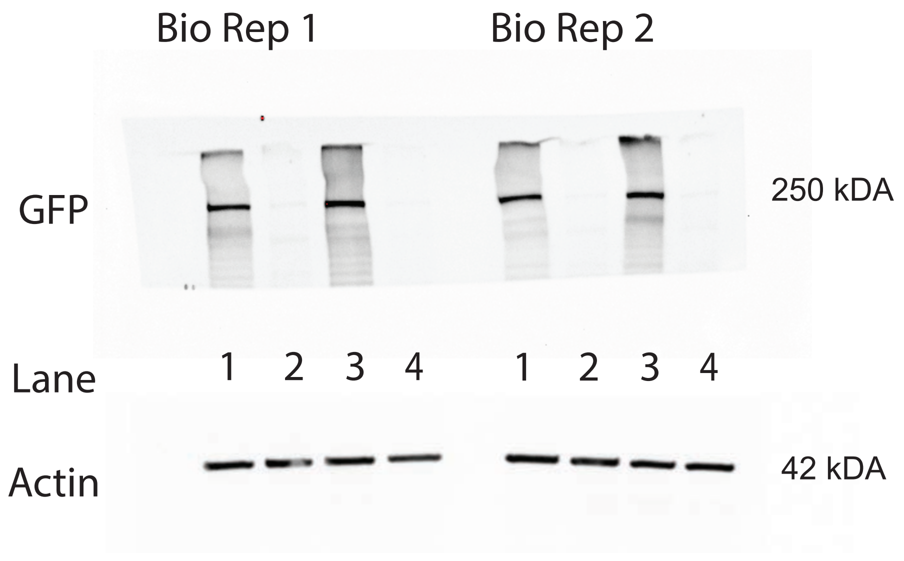

Membrane 2

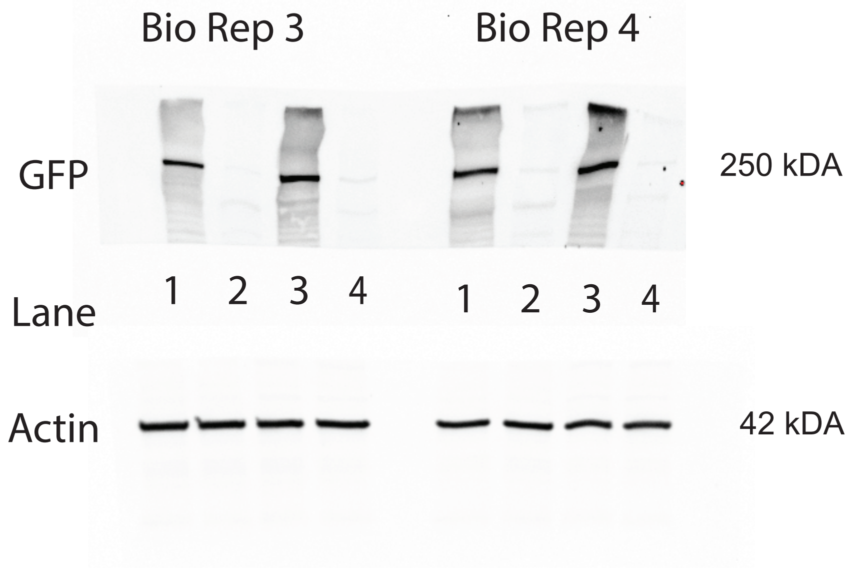

Lane/Strain+Treatment ***All samples collected at AD2

1. *eft-3p::AtTIR1(F79G)::mRuby; 3xFLAG::AID*::GFP::FASN-1*, OP50-1, vehicle, 0.1% DMSO
2. *eft-3p::AtTIR1(F79G)::mRuby; 3xFLAG::AID*::GFP::FASN-1*, OP50-1, vehicle, 5 μM 5-Ph-IAA
3. *crn-3(ok2269); eft-3p::AtTIR1(F79G)::mRuby; 3xFLAG::AID*::GFP::FASN-1*, OP50-1, vehicle, 0.1% DMSO
4. *crn-3(ok2269);eft-3p::AtTIR1(F79G)::mRuby; 3xFLAG::AID*::GFP::FASN-1*, OP50-1, vehicle, 5 μM 5-Ph-IAA

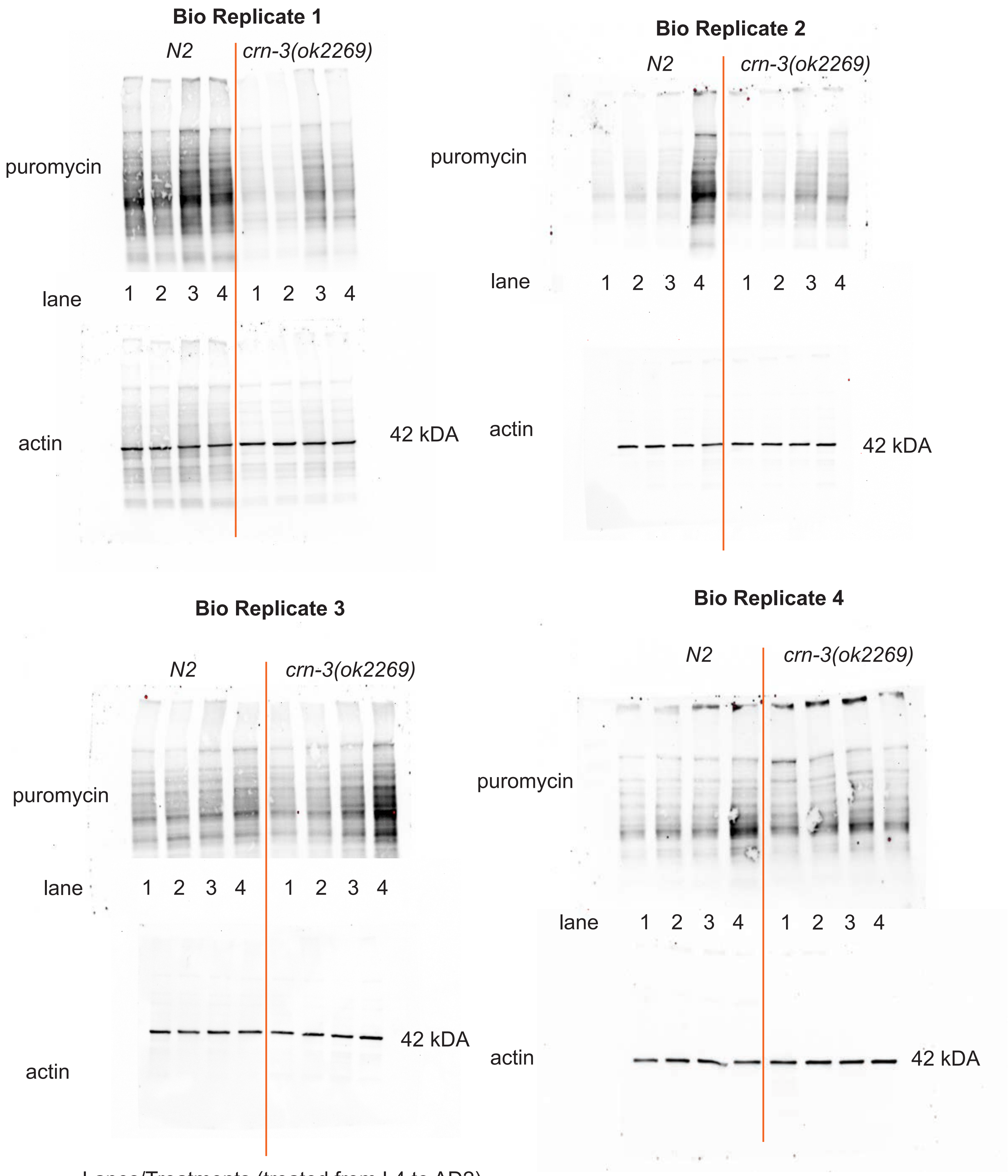

Lanes/Treatments (treated from L4 to AD2)

1. EV, Vehicle
2. EV, 4.5 mM Phenformin
3. *fasn-1* RNAi, Vehicle
4. *fasn-1* RNAi, 4.5 mM Phenformin

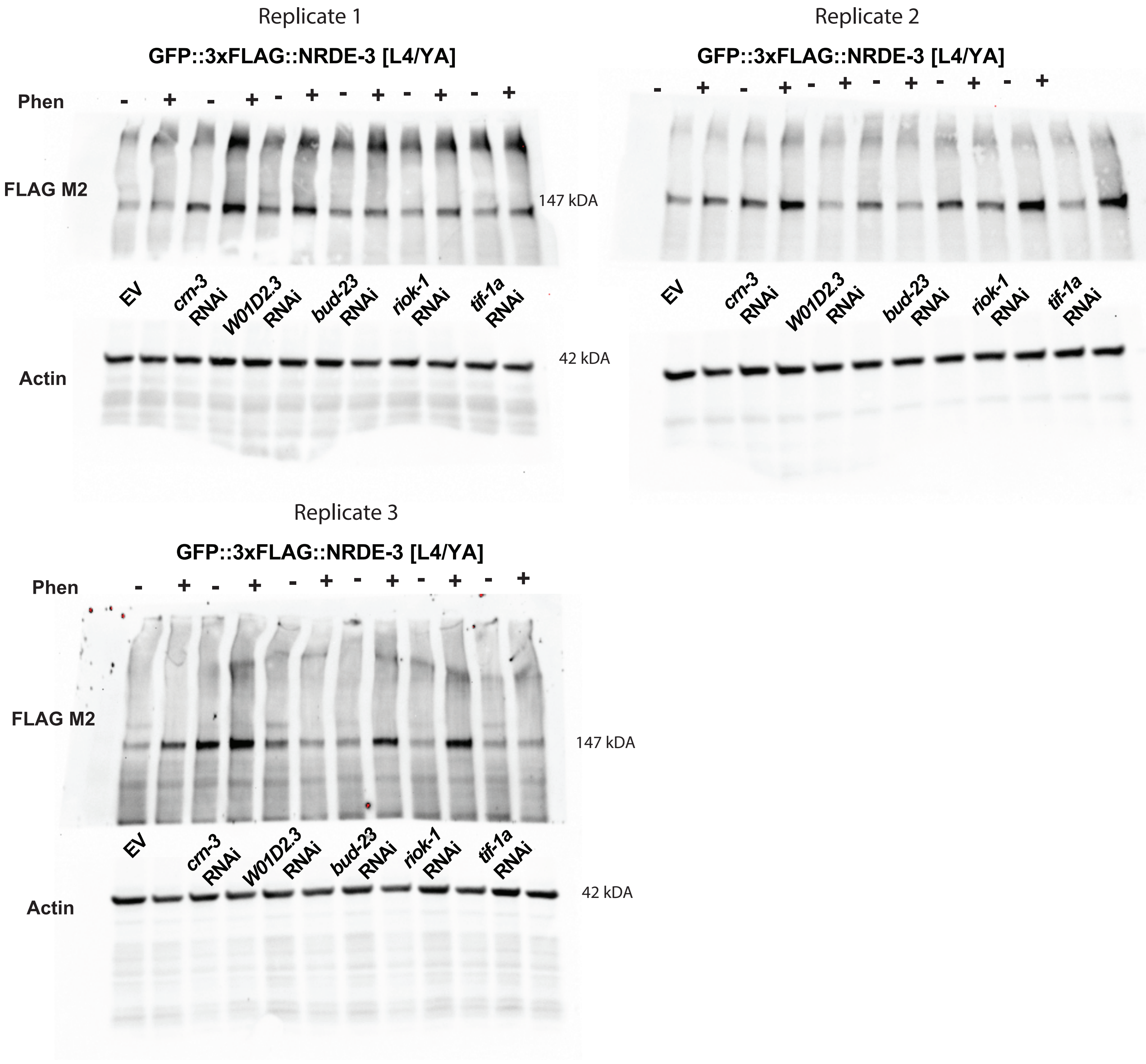
Replicate 1
GFP::3xFLAG::NRDE-3 [L4/YA]
Phen
- + - + - + - + - + - +
FLAG M2
147 kDA
EV
crn-3 RNAi
W01D2.3 RNAi
bud-23 RNAi
riok-1 RNAi
tif-1a RNAi
Actin
42 kDA
Replicate 2
GFP::3xFLAG::NRDE-3 [L4/YA]
- + - + - + - + - + - +
EV
crn-3 RNAi
W01D2.3 RNAi
bud-23 RNAi
riok-1 RNAi
tif-1a RNAi
Replicate 3
GFP::3xFLAG::NRDE-3 [L4/YA]
Phen
- + - + - + - + - + - +
FLAG M2
147 kDA
EV
crn-3 RNAi
W01D2.3 RNAi
bud-23 RNAi
riok-1 RNAi
tif-1a RNAi
Actin
42 kDA
